# Cascade Valorisation of Lemon Processing Residues (Part II): Integrated Biorefinery Design, Circular Economy, and Techno-Economic Feasibility

**DOI:** 10.3390/foods15061041

**Published:** 2026-03-16

**Authors:** Jimmy Núñez-Pérez, Jhomaira L. Burbano-García, Rosario Espín-Valladares, Marco V. Lara-Fiallos, Juan Carlos DelaVega-Quintero, Marcelo Cevallos-Vallejos, José-Manuel Pais-Chanfrau

**Affiliations:** 1 School of Agro-Industrial Engineering, Facultad de Ingenieria en Ciencias Agropecuarias y Ambientales (FICAYA), Universidad Técnica del Norte, Ibarra 100105, Imbabura, Ecuador; jlburbano@utn.edu.ec (J.L.B.-G.); rcespin@utn.edu.ec (R.E.-V.); mvlara@utn.edu.ec (M.V.L.-F.); jcdelavega@utn.edu.ec (J.C.D.-Q.); 2 Facultad de Ingenieria en Ciencias Agropecuarias y Ambientales (FICAYA), Universidad Técnica del Norte, Ibarra 100105, Imbabura, Ecuador; mcevallos@utn.edu.ec

**Keywords:** lemon biorefinery, citrus waste valorisation, cascade extraction, circular bioeconomy, pectin recovery, D-limonene, nanocrystalline cellulose, Life Cycle Assessment (LCA), Techno Economic Analysis (TEA), industrial symbiosis

## Abstract

This review examines the implementation dimensions of integrated lemon biorefinery systems, including cascade valorisation design, circular-economy integration, life-cycle assessment, techno-economic feasibility, and regulatory frameworks. Bibliometric analysis of Web of Science data (2015–2025) reveals exponential growth in citrus-biorefinery research, with lemon representing a burgeoning subset. Techno-economic assessments indicate that cascade biorefineries recovering essential oils, pectin, polyphenols, nanocellulose, and bioenergy can achieve cumulative revenues of USD 400–650 per tonne of dry peel. Whilst small-scale units (<500 tonnes per year) struggle to achieve viability, industrial simulations demonstrate Internal Rates of Return exceeding 18% at processing scales above 100,000 tonnes annually (2025 basis). Life-cycle assessments confirm environmental benefits, with greenhouse gas reductions of 60–85% relative to conventional disposal. Critical success factors include adopting green extraction technologies to preserve bioactive integrity and mitigating D-limonene inhibition in downstream anaerobic digestion. These findings establish essential oil extraction and pectin recovery as commercially mature technologies, whilst integrated multi-product lemon biorefineries remain economically promising based on techno-economic modelling and pilot-scale demonstrations, provided regulatory hurdles are effectively navigated.

## 1. Introduction

The transition from linear resource-consumption models to circular bioeconomy frameworks represents one of the most significant paradigm shifts in contemporary agro-industrial processing [1,2,3]. Within this context, the valorisation of citrus processing residues through integrated biorefinery approaches exemplifies the potential to transform waste liabilities into diversified value streams whilst simultaneously addressing environmental sustainability imperatives and economic viability constraints [4,5]. Part I of this review series established the foundational principles of cascade valorisation for lemon processing residues, examining their compositional characteristics, primary extraction pathways, opportunities for advanced products, and green extraction technologies [6]. The present study, Part II, concentrates exclusively on implementation dimensions: integrated process design, circular-economy integration, life-cycle and techno-economic assessment, regulatory frameworks, and industrial deployment pathways that determine the practical feasibility of lemon biorefineries. The successful translation of laboratory-scale extraction advances into commercially viable biorefinery operations requires systematic consideration of process integration, environmental performance optimisation, economic feasibility validation, and regulatory approval—challenges that collectively constitute the “valley of death” separating academic innovation from industrial deployment [7].

### 1.1. From Waste Management to Value Creation

The conventional approach to citrus processing waste management has historically emphasised minimising disposal costs rather than maximising value recovery, reflecting linear economic thinking in which liabilities are to be eliminated through landfilling, incineration, or low-value applications such as animal feed and composting [8]. This paradigm has generated substantial environmental burdens, including methane emissions from anaerobic decomposition in landfills, air pollution from incineration facilities, and soil contamination from uncontrolled disposal, as well as foregone economic opportunities estimated at hundreds of millions of dollars annually across the global citrus processing industry [9]. In 2020, the citrus processing sector generated approximately 15–32 million tonnes of waste worldwide, comprising peels, seeds, and pomace, collectively representing 50–55% of the processed fruit mass [10]. For 2023, global production was 23.64 million tonnes, with projections for 2024/25 of approximately 21.5 million tonnes due to adverse climatic conditions [6].

By 2010, traditional disposal methods incurred costs ranging from USD 50–100 per tonne (inflation-adjusted to 2026 estimated values: USD 70–140 per tonne) whilst simultaneously wasting resources that contain high-value bioactive compounds, structural polysaccharides, and functional materials, which command market prices exceeding USD 1000 per tonne for purified derivatives [11].

The circular bioeconomy concept fundamentally reconceptualises these waste streams as renewable feedstocks for biorefinery operations, transforming disposal costs into diversified revenue streams through systematic recovery of essential oils, polyphenols, pectin, cellulosic materials, and platform chemicals [12]. This paradigm shift aligns with international sustainability frameworks, including the United Nations Sustainable Development Goals, the European Union Circular Economy Action Plan, and national bioeconomy strategies that prioritise resource efficiency, waste minimisation, and bio-based product development [13].

Both foundational and recent techno-economic studies on citrus-derived biorefineries and waste valorisation (e.g., essential oil–pectin–biogas systems and the thermochemical conversion of orange peel waste) report positive net present values and relatively short payback periods (often 1–6 years) at suitable processing scales, highlighting the economic potential of integrated valorisation routes. However, specific revenues and payback strongly depend on plant capacity, product portfolio and market prices [14,15,16].

The environmental benefits are equally compelling, with LCA indicating that biorefinery operations reduce greenhouse gas emissions by 60–80% compared with conventional disposal practices, whilst eliminating landfill requirements and mitigating risks of soil and water contamination [17].

The compositional richness of lemon processing residues underpins the strategic rationale for integrated biorefinery approaches. Lemon peel and pulp fractions contain essential oils at 0.8–2.6% dry weight (DW), pectin at 20–30% DW, flavonoids—principally hesperidin and eriocitrin—at 1–5% DW, cellulose at 15–25% DW, and readily fermentable sugars comprising 10–20% DW [6]. This multicomponent profile directly justifies cascade biorefinery design over single-product recovery, offering four compounding advantages: (i) cumulative revenue generation that exceeds the sum of individual product values owing to process synergies and shared infrastructure; (ii) elimination of waste disposal costs and associated environmental liabilities; (iii) diversified revenue streams that buffer economic performance against volatility in any single product market; and (iv) alignment with circular economy policy frameworks that increasingly mandate resource efficiency and zero-waste targets in agro-industrial operations.

### 1.2. Circular Economy Principles in Citrus Processing

The application of circular economy principles to citrus processing systems requires systematic redesign of material flows, energy utilisation patterns, and value recovery strategies to eliminate waste generation, maximise resource productivity, and regenerate natural systems [13]. The Ellen MacArthur Foundation’s circular economy framework, widely adopted across European Union member states and increasingly influential in global policy development, establishes three foundational principles particularly relevant to citrus biorefinery design: system design to eliminate waste and pollution through preventive approaches rather than end-of-pipe solutions; keeping products and materials in use at their highest value through cascading utilisation strategies; and regenerating natural systems through nutrient recycling and ecosystem restoration [12,13].

Within citrus processing contexts, these principles manifest through multiple interconnected strategies [4]. Process integration eliminates waste generation by coupling upstream and downstream operations, in which residues from juice extraction serve as feedstocks for pectin recovery and spent peel from pectin extraction provides cellulose for nanocrystalline cellulose production. Final solid residues undergo anaerobic digestion to generate biogas, with the digestate returned to citrus orchards as an organic fertiliser, thereby closing nutrient loops [10]. Industrial symbiosis, as conceptualised in Part I [6], creates value networks in which multiple facilities exchange materials and energy, exemplified by Mediterranean citrus processing clusters, where essential oil distillation facilities supply limonene to fragrance manufacturers, pectin producers provide high-quality gelling agents to food processors, and biogas generation facilities provide renewable energy to processing operations [18,19].

Product life extension through biodegradable packaging materials derived from citrus cellulose replaces petroleum-based plastics, whilst bio-based chemical production from citrus platform molecules substitutes fossil-derived intermediates in pharmaceutical and cosmetic formulations [20].

**Figure 1:**
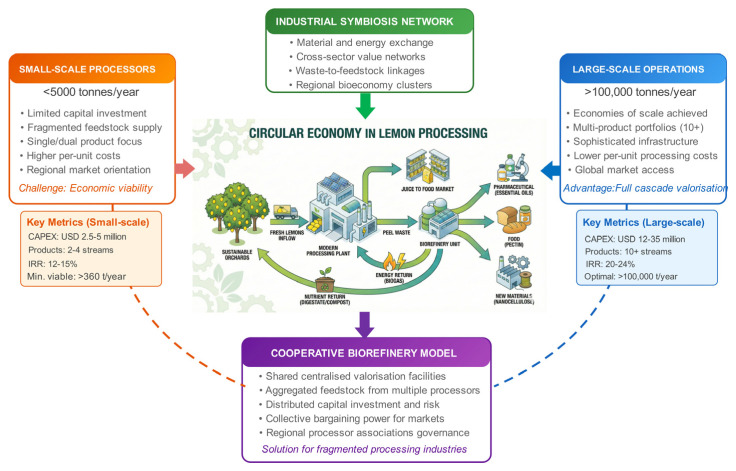
Circular Economy Integration Framework for Lemon Biorefinery Systems: Scale-Dependent Implementation Strategies and Cooperative Models (cf. Part I [6], Figure 8 for process-design scenarios). CAPEX: Capital Expenditure, OPEX: Operating Expenditure, IRR: Internal Rate of Return, NPV: Net Present Value, LCA: Life Cycle Assessment, SME: Small and Medium Enterprise, and B2B: Business-to-Business.

The economic viability of circular approaches critically depends upon achieving sufficient scale to justify capital investment in extraction and purification equipment, whilst maintaining feedstock consistency to ensure product quality standards [5,21] (Figure 1).

Circular economy integration framework for lemon biorefinery systems, illustrating scale-dependent implementation strategies and cooperative models. For orange, in 2019, small-scale processors (<5000 tonnes per year) face economic viability challenges due to limited capital investment capacity and higher per-unit processing costs [22]. Whilst large-scale operations (>100,000 tonnes per year) achieve economies of scale, enabling sophisticated multi-product biorefinery designs [22]. Cooperative biorefinery models offer a solution for fragmented processing industries by enabling shared centralised valorisation facilities, distributed capital investment, and collective market access. Industrial symbiosis networks facilitate material and energy exchange across sectors, reinforcing regional bioeconomy clusters. Key techno-economic indicators are presented for each scale category.

### 1.3. The Biorefinery Concept Applied to Lemon Waste

The biorefinery concept, initially developed for lignocellulosic biomass conversion and subsequently adapted to diverse agricultural feedstocks, provides a systematic framework for transforming lemon-processing residues into multiple value-added products through integrated chemical, biochemical, and thermochemical processing routes [2]. Analogous to petroleum refineries, which fractionate crude oil into diverse products ranging from fuels to chemicals to materials, lemon biorefineries systematically separate and transform distinct fractions of citrus waste—flavedo, albedo, seeds, and pomace—into targeted product portfolios aligned with market opportunities and profitability objectives [2,10]. The biorefinery approach fundamentally differs from single-product extraction operations by emphasising comprehensive resource utilisation, process integration, and diversified revenue streams that collectively improve economic viability whilst reducing environmental impact per unit of processed biomass [23].

Three principal biorefinery configurations have emerged for citrus waste valorisation, each offering distinct advantages and constraints depending upon available infrastructure, market access, technical capabilities, and investment capacity [10]. Biotechnological biorefineries prioritise fermentation-based conversion pathways, including anaerobic digestion for biogas production, solid-state fermentation for enzyme production, and submerged fermentation for citric acid biosynthesis, complemented by upstream extraction of inhibitory compounds, such as D-limonene, that otherwise suppress microbial metabolism [8]. Thermochemical biorefineries emphasise pyrolysis, gasification, and hydrothermal processing to convert recalcitrant lignocellulosic fractions into synthesis gas, bio-oil, and biochar products suitable for energy applications and soil amendment [24]. Integrated biorefineries, representing the most sophisticated configuration, combine extraction technologies, biochemical conversion, and thermochemical processing in optimised sequences that maximise cumulative value recovery by matching feedstock characteristics to appropriate valorisation pathways [4,25].

**Figure 2:**
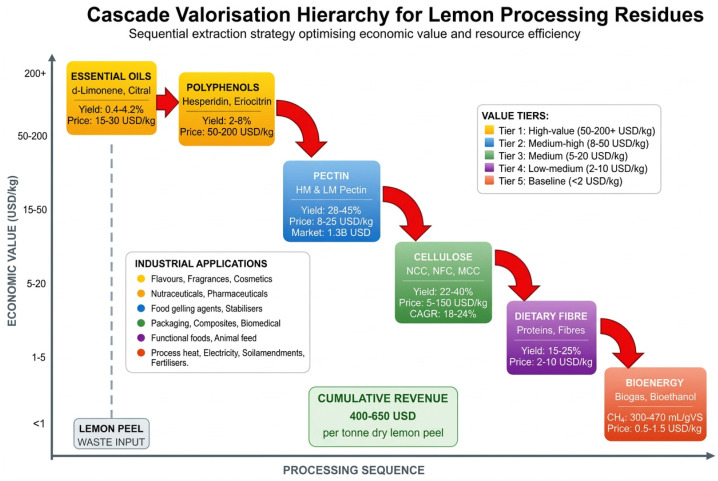
Cascade Valorisation Hierarchy for Lemon Processing Residues. EO: Essential Oil, MAE: Microwave-Assisted Extraction, UAE: Ultrasound-Assisted Extraction, PEF: Pulsed Electric Field, SFE: Supercritical Fluid Extraction, DES: Deep Eutectic Solvents, NCC: Nanocrystalline Cellulose, CNF: Cellulose Nanofibrils, MCC: Microcrystalline Cellulose, and AD: Anaerobic Digestion.

The cascade valorisation principle provides the conceptual foundation for integrated lemon biorefinery design, establishing a hierarchical framework wherein extraction processes systematically recover compounds in descending order of economic value and ascending order of processing complexity [26]. Initial process stages target readily extractable, high-value compounds such as essential oils commanding market prices of USD 15–30 per kilogram, followed by sequential recovery of pectin (USD 8–15 per kilogram), polyphenolic extracts (USD 50–200 per kilogram), and cellulosic materials (USD 5–20 per kilogram for bulk cellulose, USD 50–150 per kilogram for nanocrystalline cellulose), all in 2024 basis [25,27]. In the final stages, residual biomass is converted through fermentation or thermochemical processing into platform chemicals, biofuels, or soil amendments valued at USD 1–5 per kilogram (2020 basis) [10] (Figure 2).

This cascading approach maximises cumulative revenue whilst minimising waste generation, as each processing stage generates feedstock for subsequent operations rather than disposal-bound residues [17,28].

A comprehensive bibliometric analysis of research on lemon waste valorisation and citrus biorefineries, including network visualisation of 847 publications (2003–2025), was conducted and discussed in Part I of this review. That analysis identified the dominant thematic clusters (bioactive recovery, extraction technologies, biorefinery and value addition, bioconversion and energy, and emerging advanced materials) and revealed an increasing prominence of circular economy, Life Cycle Assessment (LCA) and techno-economic analysis (TEA) in recent years. On this basis, the present section synthesises the principal research gaps that currently constrain the transition from laboratory-scale advances to industrially implemented lemon biorefineries.

### 1.4. Research Gaps in Implementation and Commercial Deployment

Whilst Part I of this review (Section 1.5) comprehensively addressed technological gaps in extraction methodologies and product characterisation, critical knowledge deficits persist in translating laboratory-validated citrus biorefinery concepts into commercially viable industrial operations [5]. This section examines four interconnected implementation barriers that collectively impede the commercial deployment of lemon biorefinery systems: regulatory approval complexities, market development challenges, financing constraints, and supply chain integration requirements.

#### 1.4.1. Regulatory Approval Timelines and Procedural Uncertainties

The regulatory landscape governing novel biorefinery products represents a formidable barrier to market entry, characterised by prolonged approval timeframes, jurisdictional inconsistencies, and evolving scientific requirements [29]. In the European Union, novel food authorisation under Regulation (EU) 2015/2283 involves a multi-stage process that includes European Commission verification, European Food Safety Authority (EFSA) suitability assessment, scientific risk evaluation, and adoption of the final implementing act [30]. Recent comprehensive analyses of 292 novel food applications submitted between 2018 and 2024 revealed that the average duration from submission to publication of EFSA’s scientific opinion was 2.56 ± 1.19 years, with substantial variability reflecting the complexity of individual applications [31]. The validation phase alone averaged 299 ± 218 days (indicating high variability across applications), whilst scientific evaluation required 629 ± 338 days, representing approximately 21 months of technical assessment [31].

Critically, Additional Data Requests (ADRs) issued by EFSA constitute a significant source of timeline extension. Applications received an average of 2.7 ADRs, predominantly concerning production process characterisation, compositional data specifications, and toxicological assessments [31]. Applicants required an average of 130 days to respond to each ADR, with cumulative response time accounting for approximately 47% of the total scientific evaluation period [32]. These procedural delays create considerable commercial uncertainty, particularly for start-up enterprises and small-to-medium enterprises (SMEs) that must maintain operational continuity whilst awaiting regulatory decisions [33].

The September 2024 update to EFSA’s scientific guidance, effective from February 2025 and currently in force, introduced more stringent requirements for safety assessment whilst clarifying administrative procedures [30]. Although intended to enhance application quality and reduce ADRs, the guidance simultaneously raised the evidentiary threshold for novel food authorisation. For biorefinery-derived products—particularly nanocellulose derivatives and concentrated polyphenolic extracts—these enhanced requirements necessitate comprehensive toxicological dossiers that may exceed the technical and financial capabilities of smaller biorefinery operators [34].

Jurisdictional divergence compounds regulatory complexity. The United States Food and Drug Administration (FDA) operates under the *Generally Recognized as Safe* (GRAS) framework, which permits companies to make independent safety determinations without mandatory pre-market approval [35]. This regulatory architecture enables substantially faster market access but shifts the responsibility for safety assurance entirely to the applicant. The EU’s structured, centralised system provides government-backed approval but imposes extended timelines that may disadvantage European biorefinery enterprises relative to international competitors [36]. Biorefinery operators targeting global markets must therefore navigate disparate regulatory frameworks simultaneously, requiring parallel application submissions, duplicated toxicological studies, and jurisdiction-specific compliance strategies [37].

For specific lemon biorefinery products, regulatory pathways vary in their definition. Pectin (E440) benefits from established food additive status in the European Union and internationally under Codex Alimentarius (INS 440), facilitating relatively straightforward market access for conventional grades [38]. However, novel pectin derivatives with modified degrees of methoxylation or functionalised variants may warrant classification as novel foods, initiating the full EFSA evaluation process [39]. Nanocellulose crystals (NCC) derived from citrus waste occupy a particularly uncertain regulatory position, as nanomaterial-specific assessment requirements introduce additional complexity in particle characterisation, biodistribution, and long-term safety evaluation [40]. Essential oils, whilst generally holding GRAS status in the United States with established FEMA numbers, face increasing scrutiny regarding purity specifications and maximum use levels in specific applications.

The absence of harmonised international regulatory frameworks for bio-based products constitutes a systemic barrier that requires coordinated policy development. Regulatory sandbox approaches, temporary frameworks permitting controlled market access under enhanced monitoring, have been proposed to accelerate innovation whilst maintaining safety standards [33]. However, the European Union’s recent Biotech Act confirmed that novel foods will not have access to regulatory sandboxes, limiting the applicability of this innovation-enabling mechanism to the food sector [41]. Research priorities in this domain include developing standardised safety assessment protocols tailored to cascade biorefinery products, mutual recognition agreements between major regulatory jurisdictions, and enhanced pre-submission guidance mechanisms to reduce application rejection rates and ADR frequency [42].

#### 1.4.2. Market Development Challenges for Novel Bioproducts

The commercial viability of lemon biorefinery operations depends fundamentally upon establishing reliable demand channels for multiple product streams. Unlike conventional single-product manufacturing enterprises, integrated biorefineries generate diverse outputs—essential oils, pectin, polyphenolic extracts, nanocellulose, bioethanol, and digestate biofertilisers—each requiring distinct market development strategies, customer relationships, and distribution networks [29]. The absence of established markets for several novel biorefinery products constitutes a fundamental barrier to commercial investment, as industrial-scale facilities cannot achieve economic viability without secured offtake agreements [43].

Market volatility for established citrus biorefinery products introduces substantial revenue uncertainty. D-limonene prices have historically fluctuated between USD 3 and 15 per kilogram, depending on purity specifications, grade classifications, and supply and demand dynamics [44]. This price volatility reflects the interplay among citrus production seasonality, global demand cycles for essential oils, and competition from synthetic alternatives. For biorefinery operators, such price variability complicates financial projections and undermines investor confidence, particularly for facilities requiring substantial capital investment with extended payback periods [43].

Pectin markets, whilst more stable, exhibit segmentation by functional properties that influence pricing structures. High-methoxyl pectin suitable for jam and jelly applications commands prices of USD 6–10 per kilogram, whilst specialised low-methoxyl pectin for dairy applications and pharmaceutical-grade variants achieve USD 15–25 per kilogram [45]. Biorefinery operators must therefore develop extraction and purification capabilities matching specific market segment requirements, necessitating process flexibility and quality management systems that add operational complexity [46].

Novel products from lemon biorefineries face the additional challenge of creating a market rather than entering one. Nanocellulose crystals, despite demonstrated technical performance in composite materials, pharmaceutical excipients, and food structuring applications, lack established commercial channels and buyer awareness [5]. Potential industrial customers require education regarding material properties, application protocols, and comparative advantages relative to incumbent materials. This market development investment represents a high pre-commercial cost that few biorefinery developers possess the resources to undertake independently [29].

Consumer acceptance is another market development dimension that requires attention. Bio-based products derived from waste streams may encounter consumer scepticism, though the degree of resistance varies substantially across product categories. Consumer scepticism is most pronounced for direct-to-consumer edible products (e.g., functional food ingredients, dietary supplements, and fortified beverages derived from waste streams), where ‘waste origin’ perception may trigger aversion. In contrast, non-edible applications (e.g., bio-based packaging materials, industrial solvents, cleaning agents, and composite materials) derived from the same waste streams face significantly less consumer resistance, as end-users prioritise functionality and sustainability credentials over feedstock origin. Intermediate categories, such as cosmetic and personal care ingredients, occupy a middle ground where transparent communication of purity and safety standards can effectively mitigate scepticism. Effective communication strategies emphasising sustainability benefits, circular economic principles, and quality assurance mechanisms are essential for building consumer confidence. Furthermore, certification schemes—including organic certification, sustainability labels, and chain-of-custody verification—may enhance market access but impose additional compliance costs and administrative requirements.

Furthermore, whilst systematic reviews indicate that consumers express willingness to pay (WTP) premiums of 5–30% for natural or sustainably sourced products, this stated WTP does not always translate into actual purchasing behaviour, particularly in price-sensitive markets. In Business-to-Business (B2B) markets for industrial ingredients, sustainability credentials and regulatory compliance drive procurement decisions with comparatively less price elasticity, as corporate buyers increasingly integrate sustainability criteria into supply chain management. In contrast, Business-to-Consumer (B2C) markets exhibit greater price sensitivity, limited awareness of biorefinery-derived products, and scepticism towards ‘green’ claims, which collectively constrain actual premium uptake. Credible third-party certifications—including organic, Fair Trade, and carbon-neutral labels—play a critical role in bridging the gap between stated and revealed WTP by providing verifiable assurance of sustainability attributes [36,46].

A critical dimension of consumer acceptance concerns willingness to pay (WTP) a premium for eco-friendly or bio-based products, a question with direct implications for the commercial viability of lemon biorefinery outputs. Empirical evidence from consumer research suggests that WTP for green products is neither uniform nor unconditional: whilst a segment of environmentally conscious consumers consistently demonstrates readiness to pay 5–30% above conventional product prices for certified sustainable and healthy alternatives, price sensitivity remains the dominant purchasing driver for most consumers across most market segments [5,47,48,49]. This creates a fundamental tension in the positioning of biorefinery-derived products: if green processing credentials translate into higher production costs, as is frequently the case during early commercialisation phases, the premium required to achieve profitability may exceed the WTP threshold of the target consumer base.

Accordingly, market development strategies for lemon biorefinery products must carefully balance sustainability communication with competitive pricing, particularly in commodity-oriented segments such as pectin and essential oils, where synthetic or conventional alternatives provide credible low-cost substitutes.

Competition from synthetic alternatives and established supply chains presents ongoing market pressure. Synthetic D-limonene, whilst typically of lower quality for fragrance applications, offers price stability and supply security that natural variants cannot guarantee [43]. Similarly, petroleum-derived polymers continue to dominate materials markets despite growing interest in bio-based alternatives, as established infrastructure, price competitiveness, and performance predictability favour incumbent materials. Biorefinery products must therefore demonstrate clear value propositions, whether through superior functionality, sustainability credentials, or cost advantages, to displace entrenched alternatives [29].

Strategic market development priorities include establishing industry–academia–government consortia to develop market standards and specifications for novel biorefinery products, creating demonstration projects to validate product performance in commercial applications, and developing marketing infrastructure to connect biorefinery operators with potential industrial customers [50]. Long-term offtake agreements with industrial partners prior to facility commissioning can substantially de-risk biorefinery investments by guaranteeing minimum revenue streams during operational ramp-up periods [43].

#### 1.4.3. Financing Mechanisms for Biorefinery Investments

The capital intensity of integrated biorefinery installations is a fundamental barrier to commercial deployment, particularly for small- to medium-sized enterprises and enterprises in developing regions with limited access to financial markets [29]. Capital expenditure requirements for lemon biorefinery installations vary substantially with plant capacity and technological complexity, ranging from approximately USD 3.7 million for facilities processing 25,000 tonnes per year to USD 17.8 million for installations handling 400,000 tonnes per year (inflation-adjusted to 2025 values) [46]. These capital requirements exceed the financing capacity of most citrus processors, necessitating external investment that introduces additional stakeholder complexity and return expectations [43].

The fundamental misalignment between biotechnology development timelines and venture capital investment horizons constitutes a systemic financing challenge. Venture capitalists typically expect returns within 5–7-year time horizons, whilst biorefinery projects may require 3–5 years for regulatory approvals, facility construction, and operational optimisation before achieving profitability [50]. This temporal mismatch creates funding gaps at critical development junctures—often termed ‘valleys of death’—where promising technologies fail to advance due to a lack of financing rather than technical inadequacy [29].

The high capital expenditure (CAPEX) requirements for commercially profitable production, combined with relatively low profit margins in high-volume commodity production, create additional barriers to obtaining private investment [50]. Unlike digital technology investments with limited physical infrastructure requirements, biorefineries require substantial expenditures for processing equipment, storage facilities, utility infrastructure, and site development. Furthermore, the technical risks associated with scale-up from pilot to commercial scale introduce uncertainty that risk-averse investors may find unacceptable [43].

Government programmes such as the United States Small Business Innovation Research (SBIR) [51] and Small Business Technology Transfer (STTR) mechanisms partially address early-stage financing gaps, whilst the U.S. Department of Energy’s Bioenergy Technologies Office provides targeted funding for integrated biorefinery scale-up [51]. The European Union’s Horizon Europe programme and the European Investment Bank’s green financing facilities provide similar support for the development of European biorefineries. However, existing financial mechanisms have proven insufficient to fully de-risk the sector and attract the necessary private investment for widespread commercial deployment [50].

Innovative financing models warrant investigation to bridge identified funding gaps. Public–private partnerships can distribute risks between government entities seeking policy objectives and private investors seeking financial returns [29]. Green bonds and sustainability-linked financing instruments increasingly enable bio-based projects to access capital markets at favourable rates, provided projects meet defined sustainability criteria. Feed-in tariffs for biogas-derived electricity, carbon taxation schemes, waste disposal levies, and research and development tax credits collectively shape the investment landscape and can substantially improve project economics under favourable policy configurations [43].

Blended finance approaches that combine concessional public funding with commercial investment are an emerging model for biorefinery financing. Development finance institutions can provide subordinated debt or first-loss capital, reducing risk exposure for commercial lenders and enabling projects that would otherwise fail to meet conventional financing thresholds [50]. Volume guarantees—commitments by government or anchor customers to purchase minimum product quantities—can similarly de-risk revenue projections and enhance bankability [51].

An alternative low-investment entry strategy for waste generators unable to finance full biorefinery installations involves implementing only the stabilisation stage, specifically dehydration. Drying lemon processing residues to ≤10% moisture content enables: (a) a reduction in storage volume by approximately 75–80%, (b) microbial stability for extended storage periods exceeding 12 months, (c) substantially reduced transportation costs per unit of dry matter, and the (d) creation of a standardised, tradeable commodity within a Business-to-Business (B2B) model, in which centralised biorefineries purchase dried peel from multiple distributed waste generators. This model requires CAPEX investment of only 10–15% relative to a full biorefinery installation, making it accessible to small and medium processors. However, the choice of drying method must carefully balance cost-effectiveness with the preservation of volatile compounds—particularly D-limonene, which can lose 30–60% under thermal drying above 60 °C. Where essential oil recovery is envisaged as a downstream valorisation pathway, pre-drying oil extraction or the use of low-temperature alternatives (solar-assisted convective drying, vacuum drying, or freeze-drying) is strongly recommended [46,52].

Research priorities in biorefinery financing include developing standardised project finance templates tailored to biorefinery risk profiles, creating specialised investment vehicles that aggregate smaller biorefinery projects to achieve scale attractive to institutional investors, and establishing dedicated bioeconomy finance programmes within government economic development agencies [50]. Enhanced techno-economic assessment methodologies that accurately quantify project risks and returns can facilitate more informed investment decisions and reduce perceived uncertainty among potential financiers [29].

#### 1.4.4. Supply Chain Integration and Logistics Optimisation

The establishment of reliable, cost-effective feedstock supply chains represents a critical determinant of biorefinery economic viability. Unlike conventional manufacturing operations that utilise standardised industrial inputs, citrus biorefineries must manage feedstocks characterised by seasonal availability, compositional variability, high moisture content, and susceptibility to microbial degradation [52]. These characteristics impose substantial logistical complexity and storage requirements, which significantly affect operating costs and processing efficiency [51].

Seasonal feedstock availability constitutes a fundamental supply chain challenge. Citrus processing generates waste predominantly during harvest seasons, which vary by cultivar and geographical location but typically concentrate within 3–6-month annual windows [5]. Biorefinery installations designed for year-round operation must therefore either accumulate substantial feedstock inventories, incurring storage costs and quality degradation risks, or accept seasonal operational patterns that reduce capital utilisation efficiency [53]. Inventory holding costs for supporting year-round biorefinery operations can represent a substantial portion of total operating expenses, particularly for feedstocks requiring refrigerated storage or controlled-atmosphere conditions [46].

Compositional variability introduces additional supply chain complexity. Lemon processing residues exhibit variation in essential oil content, pectin yield potential, and moisture content depending on cultivar, growing conditions, harvesting practices, and post-harvest handling [44]. This variability complicates process optimisation, as extraction parameters optimised for one feedstock composition may prove suboptimal for subsequent batches. Quality variability can be partially addressed through feedstock blending strategies that homogenise inputs, but such approaches require sophisticated inventory management and quality control systems [54].

Transportation costs significantly influence biorefinery economics, particularly feedstock with high moisture content. Citrus processing residues typically contain 75–85% moisture, resulting in substantial transport costs per unit of dry matter [45]. Sensitivity analyses indicate that increasing feedstock transportation costs from USD 10 to 30 per tonne substantially raises production costs, underscoring the economic imperative to locate biorefineries near concentrated citrus processing zones [46]. Alternative strategies include establishing satellite preprocessing facilities that reduce moisture content and volume through drying or pressing operations before long-distance transport, thereby improving logistics economics at the cost of additional capital investment [54].

The dispersed geographical distribution of citrus processing facilities compounds logistics challenges. Unlike concentrated industrial feedstock sources, citrus waste generation is distributed across numerous processing facilities of varying scales [5]. Aggregating sufficient feedstock volumes to support industrial-scale biorefinery operation requires coordination across multiple suppliers, potentially involving complex contractual arrangements, quality specifications, and delivery scheduling [52]. Hub-and-spoke logistics models, in which distributed preprocessing depots consolidate and standardise feedstock before delivery to centralised biorefineries, have been proposed to address this challenge, but introduce additional handling steps and investment requirements [54].

Maintaining feedstock quality during storage is a critical operational consideration. Microbial growth at moisture content above 20% can substantially alter biomass composition and its suitability for downstream processing [51]. Essential oil content may decline through volatilisation during extended storage, reducing the value of the highest-margin product stream. Effective preservation strategies—including controlled-atmosphere storage, refrigeration, ensiling, or rapid drying—impose costs that must be balanced against the quality-maintenance benefits [52].

Critically, the choice of drying method must balance cost-effectiveness with the preservation of volatile and thermolabile compounds. Thermal drying (convective and drum drying) at temperatures exceeding 60 °C causes D-limonene volatilisation losses of 30–60%, whilst polyphenol oxidation and terpene chemical alteration further reduce the value of recovered fractions [52]. The selection of an appropriate drying strategy should therefore be guided by the intended downstream product portfolio. Where essential oil recovery is prioritised as a downstream valorisation pathway, pre-drying oil extraction is preferable, followed by drying at moderate temperatures (≤50 °C); this approach captures volatile compounds before they are lost to thermal degradation. Conversely, if the primary targets are heat-stable fractions such as pectin or cellulose, conventional hot-air drying at 60–80 °C may be acceptable without significant quality compromise. Low-temperature alternatives—solar-assisted convective drying, vacuum drying, and freeze-drying—offer superior preservation of compounds but come at higher processing costs, and their selection should be guided by the intended downstream product portfolio.

The dehydration-only stabilisation model described in Section 1.4.3 offers a practical solution to the challenge of preserving feedstock, particularly for distributed waste generators. By reducing moisture content to ≤10%, processors create a shelf-stable intermediate product that can be accumulated, traded, and transported to centralised biorefinery facilities without the quality degradation risks associated with wet biomass storage. This approach effectively decouples the temporal and spatial constraints of feedstock generation from biorefinery processing schedules, enabling year-round operation of centralised facilities whilst accommodating the seasonal nature of citrus processing [52,54]. The choice of drying conditions for this stabilisation step should be aligned with the downstream valorisation strategy: facilities targeting essential oil or polyphenol recovery should adopt milder drying protocols, whereas those focused on structural polysaccharides may employ more aggressive thermal regimes to minimise processing costs.

Circular economic principles offer opportunities for supply chain optimisation through industrial symbiosis. Co-location of biorefineries with citrus processing facilities eliminates transportation costs for fresh waste streams, whilst enabling heat integration and shared utility infrastructure [45]. Integration with complementary waste streams—such as olive processing residues in Mediterranean regions—can extend operational seasons and improve capacity utilisation, though at the cost of increased process complexity for handling heterogeneous feedstocks [5].

Research priorities for supply chain optimisation include the development of predictive models for feedstock availability and quality based on agronomic and meteorological data, design of flexible biorefinery configurations capable of adapting to feedstock variability, optimisation algorithms for logistics network design incorporating spatial distribution of feedstock sources and demand centres, and techno-economic assessment of distributed versus centralised biorefinery configurations under varying supply chain scenarios [54]. Biomass exchange platforms facilitating efficient matching between feedstock suppliers and biorefinery operators warrant investigation as mechanisms for reducing transaction costs and improving market transparency [54].

The research gaps identified in this section—regulatory approval complexity, market development challenges, financing constraints, and supply chain integration requirements—collectively represent interconnected barriers that must be addressed through coordinated action across research, industry, and policy domains. Whilst Part I established the technological foundations for lemon biorefinery operations, commercial deployment requires parallel advances in regulatory harmonisation, market infrastructure development, innovative financing mechanisms, and logistics optimisation. The following sections address these implementation dimensions in greater depth, providing the evidence base for informed decision-making regarding lemon biorefinery commercialisation.

### 1.5. Research Objectives and Scope

This review systematically examines the implementation dimensions of valorising lemon processing waste through integrated biorefinery approaches, building on the foundational principles, extraction technologies, and product opportunities established in Part I [6].

The specific objectives address critical knowledge gaps identified through bibliometric analysis and literature synthesis:

(1) Evaluate process integration strategies for cascade biorefinery configurations, analysing sequential extraction schemes, mass and energy balance optimisation, and solvent recovery systems that collectively determine operational efficiency and economic viability [2,55].
(2) Assess circular economy integration frameworks, examining industrial symbiosis opportunities, nutrient cycling pathways, and closed-loop system designs applicable to diverse geographical and institutional contexts [2,56].
(3) Synthesise LCA methodologies and findings, quantifying environmental performance across multiple impact categories and identifying process hotspots requiring intervention to achieve sustainability objectives [45,57].
(4) Analyse techno-economic assessment approaches and results, establishing capital investment requirements, operating cost structures, revenue projections, and profitability metrics for representative biorefinery configurations spanning laboratory to commercial scales [55,58].
(5) Examine regulatory compliance frameworks governing bioactive compounds, food ingredients, and nanomaterials derived from citrus waste, identifying approval pathways, toxicological requirements, and market access constraints that influence commercial deployment timelines [30,59].
(6) Review industrial implementation experiences and pilot-scale demonstrations, extracting lessons regarding scale-up challenges, operational reliability, product quality consistency, and market development that inform future biorefinery planning [19,20,60].

The scope encompasses lemon (*Citrus* × *limon*) processing residues as the primary focus, whilst incorporating relevant findings from related citrus species (orange, mandarin, grapefruit) where applicable to biorefinery design principles [2,56,61,62]. Geographic coverage emphasises Mediterranean citrus-producing regions (Spain, Italy, Turkey, Greece), South American producers (Argentina, Brazil), and emerging markets (India, China, South Africa), wherein substantial processing volumes and supportive policy environments create favourable conditions for biorefinery implementation [5,56]. Temporal scope prioritises literature published between 2020 and 2025 to capture recent technological advances, updated economic assessments reflecting current market conditions, and emerging regulatory developments, whilst incorporating seminal earlier works that established foundational concepts and methodologies [1,3,5]. The review excludes detailed coverage of extraction methodologies and green technologies, which are comprehensively addressed in Part I [6]. Instead, it emphasises downstream processing, system integration, and commercial implementation considerations essential for translating laboratory advances into viable industrial operations [1,5,63,64,65,66].

## 2. Cascade Valorisation Integration

The cascade valorisation of lemon (*Citrus × limon*) processing residues represents a paradigm shift towards resource-efficient biorefinery systems that maximise the recovery of high-value compounds while ensuring sustainability and circularity throughout the entire value chain. This strategy embodies the principle of sequential product extraction, in which each process step builds on the previous one, generating a hierarchy of products according to their market value, functionality, and downstream utility [67,68].

### 2.1. Brief Recap of Cascade Principles

**Figure 3:**
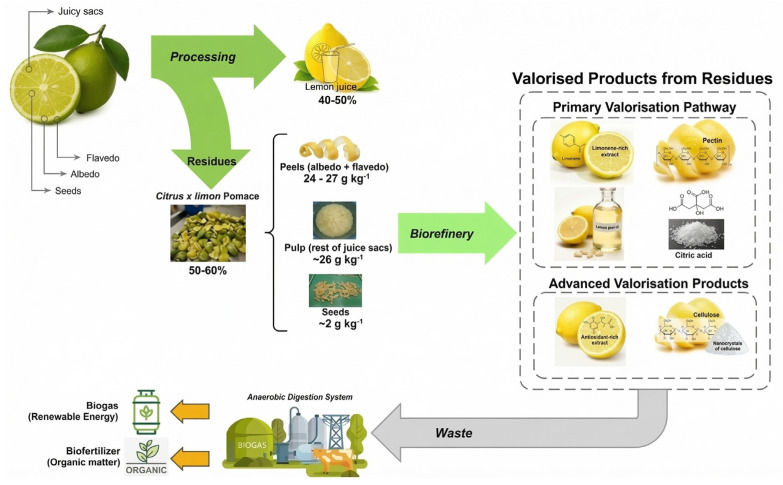
Conceptual framework for cascade valorisation of lemon processing waste within a circular biorefinery model. Sequential extraction and conversion pathways for maximising economic value and resource efficiency through hierarchical recovery of essential oils, pectin, bioactive compounds, dietary fibres, cellulose-derived materials, and bioenergy from lemon residues.

Cascade valorisation extends the core tenets of the circular bioeconomy established in Part I (Section 1.3), emphasising the closed-loop recovery of co-products and by-products, thereby discouraging linear ‘end-of-life’ disposal and promoting the continuous utilisation of biomass resources [69]. At the core of this approach is the methodological separation of lemon biomass into discrete, functionally distinct fractions—peels, seeds, pomace, aqueous effluent, and essential oils—each of which is prepared for targeted downstream processing (Figure 3).

Several guiding principles underpin cascade valorisation:

- **Minimisation of Waste**: Each process stage is designed to exhaustively convert waste streams into valuable inputs for subsequent operations, thereby reducing environmental burden and landfill dependency [67].
- **Sequential Product Hierarchy**: Products are extracted in order of highest added value, beginning with essential oils and pectin, and continuing with polyphenols, bioactive fibres, protein isolates, and finally, bioenergy intermediates [2].
- **Process Integration**: Where possible, operations are integrated to share utilities (heat, water, enzymes) and infrastructure, maximising efficiency and reducing costs [70,71].
- **Life Cycle and Sustainability**: The sustainability of the cascade is rigorously monitored through Life Cycle Assessment (LCA) and techno-economic modelling, enabling the selection of optimal product portfolios and minimising energy and material consumption [57].

Recent advances have led to the adoption of green, intensified extraction methods, including ultrasound-assisted [72], enzyme-assisted [73], and microwave-assisted [74] processes. These enable the selective recovery of target compounds at lower environmental and economic cost. Notably, such approaches also enhance product purity and functionality, thereby securing their competitive position in high-value markets.

### 2.2. Integration Considerations for Industrial Systems

The operational viability of cascade biorefineries is closely tied to the integration of upstream and downstream processing steps, a theme of growing significance in the literature [10,75]. Modular, interoperable process designs enable the sharing of utilities (such as energy, water, and enzyme substrates) across extraction steps, thereby reducing material waste and operational costs.

Critical components of process integration include:

- **Multi-Step Extraction Platforms**: Automated extraction units capable of operating in series—oil separation, pectin recovery, polyphenol isolation—provide scalability and flexibility, adapting to variations in feedstock and market demand [2,62].
- **Utility Recovery Systems**: Closed-loop water and heat recovery schemes lower overall environmental impact [76,77], while the recirculation of effluents and by-products supports downstream fermentation and nutrient recycling [78]. Quantitative assessments indicate that heat integration in cascade biorefineries—for instance, using waste heat from steam distillation for downstream drying operations—reduces overall energy consumption by 25–40% relative to standalone operations [70,71]. Regarding water efficiency, closed-loop recycling systems incorporating membrane filtration and process water recirculation have been reported to achieve water reuse rates of 60–80%, reducing freshwater consumption from 12–15 L per kg of dry peel in open-circuit operations to fewer than 5 L per kg. Similarly, ethanol recovery via distillation in pectin extraction processes achieves solvent recovery rates of greater than 90%, reducing solvent procurement costs by approximately 70–80% relative to single-use operation.
- **Life Cycle and Techno-Economic Modelling**: Rigorous monitoring and modelling of energy flows, emissions, and economic returns ensures that process integration delivers both environmental and financial sustainability [45].

Recent advances in digital process control and data analytics further strengthen integration prospects, enabling real-time optimisation of extraction parameters and product portfolio management aligned with fluctuating demand [2,77,79,80].

### 2.3. Process Flow Configurations

The concept of a cascade or staged biorefinery is fundamental for maximising biomass value. Instead of optimising the production of a single product, this approach seeks to build a diversified portfolio, starting with the highest-value products and sequentially using residual fractions to generate lower-value products. This model improves the economic sustainability of the process by creating multiple revenue streams and increases environmental sustainability by minimising waste generation [81,82].

#### 2.3.1. Comparison of Schemes: “Extract-First” vs. “Hydrolyse-First”

Based on the literature, two central conceptual schemes can be distinguished for citrus residue biorefining:

**Extract-First**: This is the most researched and considered the most economically viable approach. In this scheme, high-value compounds (essential oils, pectin, phenolic compounds) are extracted in the initial stages using selective technologies, such as ultrasound-assisted extraction or supercritical fluid extraction [83]. The residual biomass then serves as the feedstock for the second stage, generating higher-volume, lower-value products such as bioethanol or biogas. The need to extract D-limonene to prevent microbial inhibition in fermentation is a clear example that validates the logic of this approach [84].

**Hydrolyse-First**: In this approach, the entire biomass is hydrolysed to release all fermentable sugars. Subsequently, the valuable compounds would be attempted to recover from the process streams. Although conceptually possible, this route poses greater challenges for purifying and separating high-value compounds, which may degrade or be diluted during hydrolysis [85].

#### 2.3.2. Intersectoral Integrated Model for Citrus Valorisation

The Intersectoral Integrated Model for Citrus Valorisation proposes a biorefinery orientated towards sustainability through the cascade valorisation of lemon by-products (*Citrus* × *limon*). This approach optimises the sequential utilisation of solid residues—peel, albedo, and seeds—by prioritising the recovery of high-value-added compounds, such as essential oils (e.g., limonene, citral) and pectin with pharmaceutical and food applications. Subsequently, the residual streams are directed to the production of bulk commodities or to biotechnological processes, including fermentation for citric acid production and alkaline extraction of high-purity α-cellulose [71,86]. This model enables the closure of material and energy cycles, reducing the environmental footprint of the citrus industry. Technological integration is crucial. De-oiled and depectinised residues are converted into feedstock for advanced biotechnological processes, including citric acid extraction or alkaline extraction to obtain high-purity α-cellulose [20,27]. This model enables the closing of material and energy cycles, reducing the environmental footprint of the citrus industry. This cellulose holds excellent potential for producing composite materials [3], advanced biofuels [87], or serving as a base for nanotechnology [88,89].

**Figure 4:**
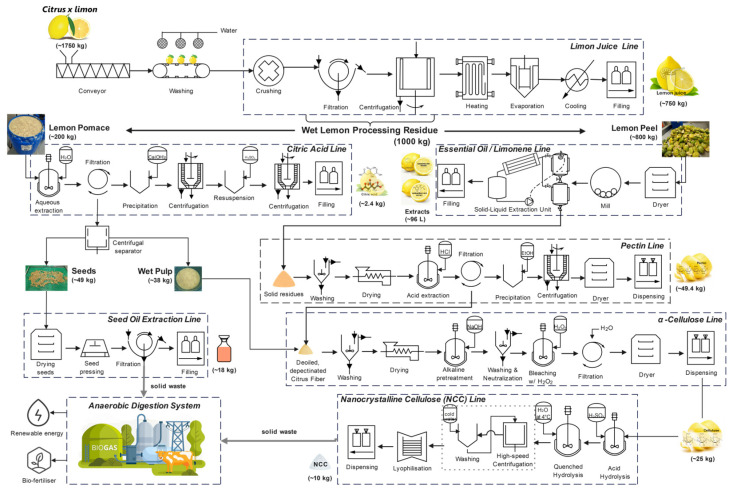
Process Diagram of an integrated lemon biorefinery under an intersectoral model.

Figure 4 illustrates the cascade valorisation of citrus residues, integrating technological pathways that maximise economic value while minimising environmental impact. Optimising this configuration requires a rigorous Life Cycle Assessment (LCA) and a detailed Techno-Economic Analysis (TEA) [90].

These analyses should focus on key variables such as energy efficiency, extraction yield, and separation and purification costs [91,92]. The successful implementation of this model depends on its adoption in regions with a strong existing agro-industrial infrastructure [29], which enables synergy with sectors such as the sugar or paper industries to reinforce industrial circularity.

#### 2.3.3. Economic Hierarchy and Recovery Logic

The intrinsic diversity of lemon residues offers a rich palette of value-added compounds, each possessing distinct physico-chemical properties and economic appeal [63]. Cascade valorisation establishes a logical sequence for their recovery [93,94], prioritises the following hierarchical stages:

i. **Essential Oils and Volatiles**: The initial recovery of essential oils is technically mandatory to prevent microbial inhibition in subsequent fermentation steps. Economically, this fraction represents the first high-value stream (USD 15–30 kg^−1^), capturing volatile revenue prior to thermal degradation steps [10,95,96].
ii. **Pectin**: Pectin recovery constitutes the **economic backbone** of the biorefinery due to its substantial global market volume (approximately 60,000 metric tonnes annually, valued at USD 1.1–1.3 billion in 2024, with projections reaching USD 2.1 billion by 2034 at a 5.5–7.0% CAGR). Its extraction logic involves using the de-oiled solid residue to amortise the operational costs (OPEX) of the acid/enzymatic hydrolysis stage. Cascade valorisation enhances pectin’s techno-economic feasibility by integrating upstream oil separation with extraction and downstream polyphenol recovery [97,98].
iii. **Polyphenols and Flavonoids**: Targeting high-purity polyphenols shifts the biorefinery into the **pharmaceutical value tier**. While volumes are lower, the unit value is significantly higher, justifying the investment in purification technologies (e.g., membrane filtration) after bulk pectin removal [99,100].
iv. **Cellulose and Nanocellulose**: After pectin extraction, the remaining lemon biomass converts the final solid residue into Nanocellulose, creating a value leap (USD 50–150 kg^−1^) compared to traditional low-value routes like animal feed or compost, maximising the total revenue per tonne of processed fruit [3,20].
v. **Lignocellulosic Biomass Valorisation**: Following the removal of limonene, pectin, polyphenols, and cellulose derivatives, the residual solid matrix—composed mainly of cellulose, hemicellulose, and lignin—is well-suited for biotechnological upgrading. Solid-state fermentation (SSF) enables the use of specialised fungi (e.g., *Trichoderma reesei*, *Aspergillus niger*) and yeasts (*Saccharomyces cerevisiae*, *Candida utilis*) to produce industrially relevant enzymes (cellulases, xylanases) and single-cell protein (SCP) for food, feed, or biocatalytic applications [73,101].
vi. **Bioenergy, Biochar, and Soil Amendments**: The final valorisation step transforms recalcitrant residues (pomace, seeds, effluent solids) through anaerobic digestion [102], pyrolysis [87], and composting [103], providing bioethanol [23], biohydrogen [104], and biofertilisers [105] that closes the resource recovery loop.

The comparative assessment of citrus biorefinery configurations requires a systematic evaluation across multiple performance dimensions, including technical feasibility, economic viability, environmental sustainability, and implementation complexity [106,107].

- Firstly, economic viability is strongly correlated with product portfolio diversification, whereby biorefineries recovering four or more distinct product streams achieve substantially higher profitability metrics than single-product or dual-product configurations [14,70,108,109]. This finding underscores the fundamental importance of cascade valorisation approaches that systematically extract multiple value fractions rather than targeting isolated high-value compounds [26].
- Secondly, capital investment requirements exhibit non-linear scaling relationships, with per-tonne processing capacity costs declining substantially as annual throughput increases from small scale (~5000 tonnes per year) through intermediate scale (10,000–50,000 tonnes per year) to large scale (>100,000 tonnes per year), suggesting that cooperative models enabling smaller processors to share centralised valorisation facilities merit serious consideration in regions characterised by fragmented processing industries [110,111].
- Thirdly, environmental performance metrics consistently favour integrated biorefinery configurations over conventional disposal practices across all impact categories assessed, including global warming potential, eutrophication potential, acidification potential, and resource depletion indicators, with emission reductions ranging from 60 to 85%, depending on specific processing schemes and energy sources employed [17].
- Fourthly, technology readiness levels remain distributed across a broad spectrum, with essential oil extraction and pectin recovery having achieved commercial maturity (TRL 8–9), enabling immediate implementation, whilst advanced products, including nanocrystalline cellulose, industrial enzymes, and platform chemicals, remain predominantly at pilot scale (TRL 5–7), requiring additional development before routine commercial deployment becomes feasible [22]. This technology maturity distribution suggests phased implementation strategies in which initial biorefinery configurations emphasise established products to secure reliable revenue streams, whilst progressively incorporating emerging products as technical risks diminish and markets mature [4,112].
- Fifthly, sensitivity analyses consistently identify feedstock cost, product market prices, and processing scale as the three parameters exerting dominant influence on profitability metrics, with secondary importance attributed to energy costs, solvent recovery efficiency, and labour requirements [2,14,113].

This parametric hierarchy indicates that successful biorefinery implementation critically depends on securing stable, low-cost feedstock supplies through long-term contracts with juice processors, establishing reliable market access for multiple products to mitigate price volatility, and achieving sufficient processing scale to justify capital investment in sophisticated extraction and purification equipment [10].

Finally, geographical analysis reveals that successful commercial implementations concentrate in regions combining substantial citrus processing volumes, supportive regulatory environments, established markets for biorefinery products, and available technical expertise, suggesting that technology transfer to emerging markets requires parallel development of institutional capacity, market infrastructure, and policy frameworks rather than equipment transfer alone [8].

## 3. Circular Economy Integration: Closing the Loop

The implementation of circular economy principles within lemon biorefinery systems represents a fundamental shift toward the sustainable valorisation of citrus processing residues [2]. This approach aligns with the European Green Deal and Circular Economy Action Plan, which prioritise the recovery of bio-based materials from agro-industrial by-products [114]. The circular economy framework transforms linear ‘take-make-dispose’ production models into closed-loop systems wherein waste streams become valuable feedstocks for subsequent processes, thereby minimising environmental burdens whilst maximising economic returns [47]. In the context of a lemon biorefinery, circular economy integration extends beyond the primary valorisation of essential oils, pectin, and bioactive compounds to encompass the comprehensive utilisation of all residual fractions through secondary bioconversion processes, particularly anaerobic digestion for bioenergy recovery and nutrient cycling [115].

### 3.1. Anaerobic Digestion for Final Residues

Following the cascade extraction of high-value compounds, substantial quantities of de-pectinised biomass remain. Anaerobic digestion (AD) emerges as the most technically feasible technology for valorising these final residues, converting organic matter into biomethane. However, the specific challenge in lemon processing stems from the potent antimicrobial properties of residual D-limonene [116]. The anaerobic digestion process converts complex organic matter into biomethane through a series of sequential biochemical transformations, including hydrolysis, acidogenesis, acetogenesis, and methanogenesis, mediated by consortia of anaerobic microorganisms operating under oxygen-free conditions [117]. D-limonene, which constitutes 90–95% of citrus essential oils, exhibits toxic effects on slow-growing methanogens via membrane disruption, potentially causing process instability and reduced biogas yields at concentrations above the threshold [23,115,118].

Research demonstrates that D-limonene concentrations exceeding 423 mg·kg^−1^ vs. progressively inhibit methanogenic archaea, with complete process failure occurring at concentrations approaching 669 mg·kg^−1^ under batch conditions [119]. Consequently, successful implementation requires either exhaustive upstream extraction of essential oils (to achieve both economic and biological stability) or the use of two-stage AD systems. In such configurations, the first stage (acidogenesis) operates at thermophilic temperatures to facilitate the volatilisation and stripping of residual limonene, protecting the sensitive methanogens in the subsequent mesophilic stage [116]. This integration exemplifies the circular economy principle: removing a high-value inhibitor (limonene) is a prerequisite for efficient energy recovery (biogas).

Two-stage AD systems have demonstrated superior performance compared to single-stage configurations when processing citrus waste containing residual D-limonene [116]. In these systems, the first reactor operates as a hydrolytic and acidogenic unit at thermophilic temperatures (typically 55 °C), facilitating rapid degradation of complex carbohydrates whilst simultaneously enabling partial volatilisation and separation of hydrophobic D-limonene through phase partitioning [117]. The liquid effluent from the primary reactor, depleted of lipophilic inhibitory compounds, subsequently enters a second methane formation [116]. Research on Colombian citrus processing wastes achieved methane concentrations of approximately 60% by volume using a two-stage anaerobic digestion process, with biodigestion commencing after a 19-day stabilisation period for the upflow anaerobic sludge blanket (UASB) methanogenesis reactor [116].

Pilot-scale investigations employing semi-continuous feeding regimes have quantified the optimal operational parameters for the anaerobic digestion of citrus peel. Under mesophilic conditions (42 °C), the maximum specific methane yield was attained at an organic loading rate of 1.0 g·L^−1^·day^−1^ of volatile solids and an essential oil supply rate of 47.6 mg·L^−1^·day^−1^, whilst partial inhibition manifested at an organic loading rate of 1.98 g·L^−1^·day^−1^ of volatile solids [120]. Thermophilic digestion exhibited greater sensitivity to D-limonene toxicity, with cumulative methane production (0.12 L·g^−1^ of volatile solids) representing only 25% of the yield achieved under mesophilic conditions (0.46 L·g^−1^ of volatile solids), suggesting that mesophilic operation provides enhanced process stability for citrus waste digestion [120].

Advanced reactor technologies have been developed to overcome D-limonene-mediated inhibition whilst maximising biogas yields. Membrane bioreactor systems, wherein methanogenic archaea are encapsulated within hydrophilic membranes whilst freely suspended hydrolytic and acidogenic bacteria degrade citrus substrates externally, have achieved methane productions of 0.33 Nm^3^·kg^−1^ volatile solids—equivalent to approximately 73% of theoretical yields—at organic loading rates of 3 kg·m^−3^·day^−1^ of volatile solids [121,122]. This configuration effectively protects sensitive methanogens from lipophilic inhibitors whilst maintaining nutrient accessibility through membrane permeability. Effluent recirculation strategies in two-stage systems have similarly demonstrated efficacy in mitigating pH fluctuations and improving overall process stability, as the methanogenic effluent provides buffering capacity to the acidogenic reactor [123].

Integrating anaerobic digestion into lemon biorefinery schemes generates multiple value streams beyond methane production. The digestate byproduct is a nutrient-rich organic fertiliser that contains nitrogen, phosphorus, potassium, and micronutrients in stabilised forms readily assimilable by crops, thereby reducing reliance on synthetic fertilisers and contributing to sustainable agricultural practices [116,117]. The biogas produced can be upgraded to biomethane standards and utilised for on-site heat and electricity generation through combined heat and power systems, substantially reducing the biorefinery’s external energy requirements and operational costs [118]. Economic analyses indicate that implementing anaerobic digestion of post-extraction citrus residues can yield biogas production of 45 m^3^ per tonne of citrus waste processed, alongside recovery of 8.4 L of D-limonene when flash steam explosion pretreatment is employed to extract residual essential oils before digestion [124,125].

The operational parameters discussed above demonstrate the critical importance of reactor configuration and operating conditions in determining the success of anaerobic digestion systems for lemon processing residues. To facilitate comparative evaluation of these technological alternatives, Table 1 consolidates the optimal operational parameters reported across recent pilot-scale and semi-continuous investigations.

This comparative framework enables biorefinery designers to select the most appropriate digestion configuration based on feedstock characteristics, process stability requirements, and methane production targets, whilst accounting for the specific inhibitory effects of residual D-limonene in post-extraction citrus biomass.

### 3.2. Life Cycle Assessment (LCA) Considerations

The LCA methodology provides a comprehensive quantification of environmental burdens and impacts associated with lemon biorefinery operations across the entire value chain, from agricultural production through processing, valorisation, and end-of-life management. The LCA framework, governed by the ISO 14040:2006 and ISO 14044:202006 international standards [126,127], enables systematic identification of environmental hotspots, comparative evaluation of alternative valorisation scenarios, and guidance for process optimisation to improve sustainability performance [45,68].

**Figure 5:**
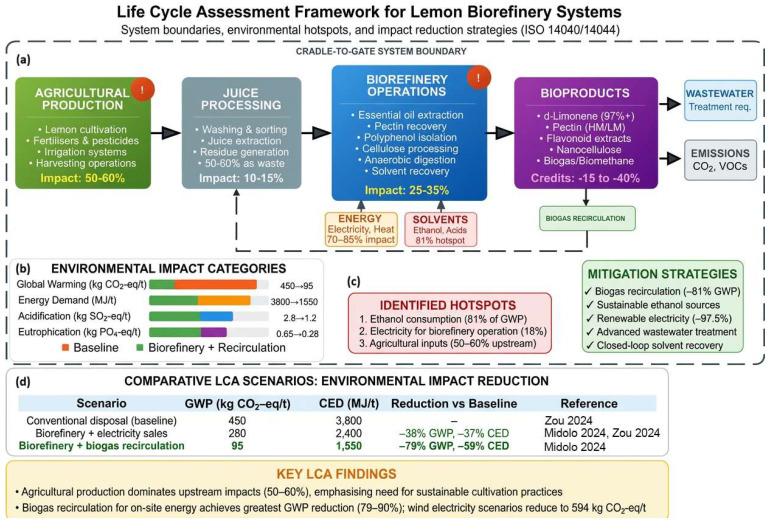
Life Cycle Assessment (LCA) framework for integrated lemon biorefinery systems. (**a**) System boundaries encompass agricultural production, juice processing, biorefinery operations, product distribution, and end-of-life management, with material and energy flows indicated across life-cycle stages. (**b**) Contribution analysis of environmental impact categories for baseline (conventional disposal), biorefinery with external biogas sale, and biorefinery with complete biogas recirculation scenarios. (**c**) Sensitivity analysis demonstrating the influence of key parameters (energy source, transportation distance, and extraction efficiency) on global warming potential. (**d**) Comparative environmental performance across valorisation scenarios, illustrating reductions in global warming potential (79–90%), cumulative energy demand (59–78%), and eutrophication potential (57–72%) achievable through circular biorefinery integration. LCA methodology complies with ISO 14040:2006 and ISO 14044:2006. GWP: Global Warming Potential, CED: Cumulative Energy Demand, EP: Eutrophication Potential, AP: Acidification Potential, and ISO: International Organisation for Standardisation [128].

Recent LCA investigations of citrus biorefinery systems have revealed that environmental impacts vary considerably depending on the selection of functional units, allocation methodology, system boundaries, and the specific valorisation pathways implemented [68] (Figure 5).

In the context of lemon processing, agricultural production consistently accounts for the dominant share of overall environmental impacts across multiple categories. Studies on Argentine lemon production chains demonstrate that agrochemicals—including synthetic fertilisers, pesticides, and herbicides—account for more than 60% of impacts across eleven of twelve evaluated categories, whilst agricultural activities contribute over 50% of total burdens even when downstream industrial processing is considered [129]. For processed lemon derivatives, such as concentrated juice, essential oil, and dehydrated peel, agriculture remains the primary source of impact, followed sequentially by natural gas consumption and electrical energy utilisation during industrial operations [129]. These findings underscore the critical importance of implementing sustainable agricultural practices, including precision fertilisation, integrated pest management, and conservation tillage, to achieve meaningful reductions in the environmental footprint of lemon-derived products [130].

The implementation of biorefinery approaches that incorporate circular-economy principles does not automatically guarantee superior environmental performance compared to conventional processing schemes. Comparative LCA analyses of baseline lemon processing (producing essential oil, concentrated juice, and dehydrated peel) versus biorefinery scenarios that generate additional products, such as ethanol, purified D-limonene, and biogas, through various recirculation schemes have yielded nuanced results [129,130]. The most environmentally favourable scenario identified was complete recirculation of biogas for on-site electricity generation, which demonstrated the lowest impacts in eight of twelve categories analysed, including global warming potential, cumulative energy demand, and acidification potential [129,130]. This configuration capitalises on renewable energy substitution effects, displacing fossil-fuel-derived electricity from the grid whilst simultaneously reducing waste-management burdens.

Allocation methodology significantly influences LCA outcomes for multi-product biorefinery systems. Economic allocation, in which environmental burdens are apportioned among co-products in proportion to their market values, has been widely employed in citrus LCA studies [68]. For instance, in combined pectin and essential oil extraction systems, economic allocation may attribute 84.79% of impacts to pectin (the primary product with the highest market value), 13.24% to essential oil, and 1.97% to juice concentrate. Alternative allocation approaches based on mass, energy content, or system expansion yield divergent results and interpretations, highlighting the necessity for transparent methodological reporting in comparative LCA studies.

Process-specific LCA investigations have identified key environmental hotspots within lemon biorefinery operations. Energy-intensive unit operations—particularly drying, distillation, and extraction processes—constitute major contributors to global warming potential and cumulative energy demand [68]. Conventional pectin extraction, employing elevated temperatures and extended reaction times with mineral acids, generates substantial environmental burdens. In contrast, the adoption of green extraction technologies, such as microwave-assisted, ultrasound-assisted, and subcritical water extraction, can reduce energy consumption by 30–60% while maintaining or improving extraction yields [47]. Solvent selection and recovery strategies similarly influence environmental profiles; systems incorporating closed-loop solvent recycling demonstrate markedly improved performance across terrestrial ecotoxicity, human toxicity, and resource depletion categories [45].

Recent LCA studies on citrus biorefineries that incorporate D-limonene extraction, anaerobic digestion, and digestate utilisation reveal environmental benefits relative to baseline scenarios involving landfilling or incineration of processing waste. A gate-to-gate assessment of combined D-limonene and pectin production from orange peel waste, followed by anaerobic digestion of residual biomass, identified electricity consumption and thermal energy requirements as dominant contributors to environmental impacts, together accounting for 70–85% of burdens across categories, including climate change, eutrophication, and photochemical oxidation [45]. Scenario analyses demonstrate that biogas recirculation for on-site energy generation reduces global warming potential by 81–89% and cumulative energy demand by 58–61% compared with conventional waste-disposal pathways [131]. Furthermore, the utilisation of digestate as an organic fertiliser mitigates the eutrophication potential by partially displacing synthetic nitrogen and phosphorus fertilisers, which carry substantial production-related environmental burdens [111].

Transportation distances between lemon processing facilities and biorefinery installations are a critical factor influencing the environmental and economic viability of both. Life cycle analyses incorporating spatial considerations emphasise the importance of industrial symbiosis arrangements in which biorefinery units are co-located with, or proximate to, citrus juice extraction plants, thereby minimising transportation-related emissions, costs, and logistical complexities [68]. The regional concentration of citrus processing activities, particularly evident in Mediterranean zones such as Sicily, southeastern Spain, and California, facilitates the establishment of integrated biorefinery clusters capable of achieving economies of scale while reducing the environmental burdens associated with biomass logistics [45]. The preceding discussion of LCA methodologies and allocation approaches underscores the significant environmental variability inherent to different biorefinery configurations.

Table 2 quantifies these differences by comparing key impact categories across three representative scenarios: conventional baseline processing, biorefinery with external biogas sales, and an integrated biorefinery with complete biogas recirculation for on-site energy generation.

The data presented illustrate the substantial environmental benefits achievable through the implementation of a circular economy, particularly in terms of reducing global warming potential and cumulative energy demand, while simultaneously highlighting the relative performance of alternative valorisation strategies across multiple environmental dimensions.

### 3.3. Techno-Economic Analysis

**Figure 6:**
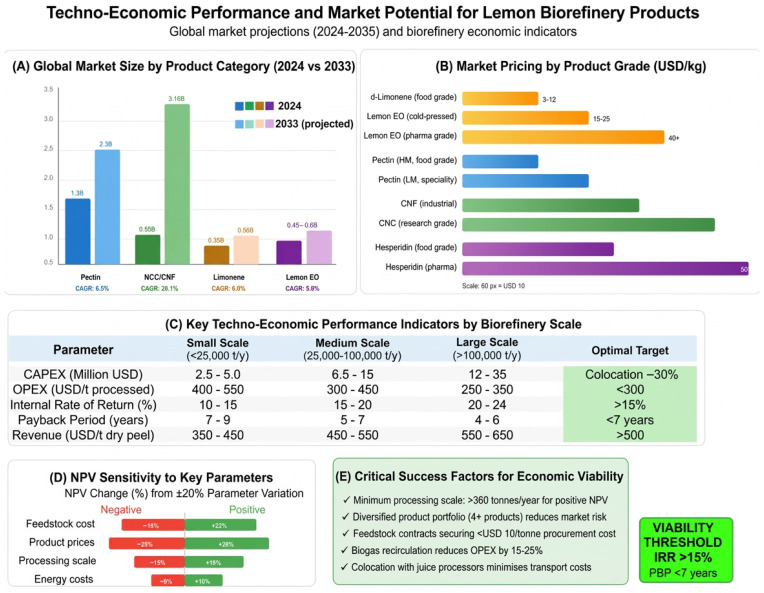
Techno-economic analysis framework for lemon biorefinery configurations at varying scales. (**A**) Process flow diagram illustrating mass and energy balances for integrated essential oil, pectin, polyphenol, and bioenergy recovery from lemon processing residues. (**B**) Capital expenditure (CAPEX) and operating expenditure (OPEX) breakdown by unit operation, demonstrating the relative contributions of extraction, purification, solvent recovery, and utility systems. (**C**) Scale-dependent economic indicators, including internal rate of return (IRR), net present value (NPV), and payback period as functions of annual processing capacity (25,000–400,000 tonnes per year). (**D**,**E**) Sensitivity analysis tornado diagram identifying key economic drivers: feedstock cost, product market prices (particularly D-limonene and pectin), solvent recovery efficiency, and energy costs. Economic parameters assume a 10-year project lifetime, an 8% discount rate, and 330 operating days per year. CAPEX: Capital Expenditure, OPEX: Operating Expenditure, IRR: Internal Rate of Return, NPV: Net Present Value, TEA: Techno-Economic Analysis, PBP: Payback Period, and CHP: Combined Heat and Power.

Techno-Economic Analysis (TEA) provides a quantitative evaluation of financial viability, capital requirements, operating expenditures, and profitability of lemon biorefinery implementations at commercial scale. Comprehensive TEA studies integrate process engineering considerations—including mass and energy balances, equipment sizing, and operational parameters—with economic modelling incorporating capital cost estimation, cash flow analysis, sensitivity assessments, and profitability indicators such as net present value, internal rate of return, and minimum selling price [113] (Figure 6).

The key assumptions underlying the reported Figure 6 are:

- **Price assumptions**: Product prices are based on 2024–2025 market data for each product category (essential oils: USD 15–30/kg; pectin: USD 10–15/kg; polyphenolic extracts: USD 50–200/kg; nanocellulose: USD 5–15/kg at commercial scale). Sensitivity analyses (Figure 6D,E) demonstrate the impact of ±20% price fluctuations on IRR and NPV.
- **Market volatility**: The cumulative revenue range (USD 400–650 per tonne) already encompasses scenarios from conservative to optimistic market conditions. D-limonene price volatility (historical range: USD 3–15/kg) represents the primary source of revenue uncertainty, as noted in Section 4.4.
- **Plant lifetime**: Economic parameters assume a 10-year project lifetime, an 8% discount rate, and 330 operating days per year (as stated in the Figure 6 caption). Payback periods of 5–9 years fall within this assumed project horizon.

Noting that the projections shown in Figure 6 are derived from process simulations and literature-reported pilot/industrial data, not from a single operational facility, and that actual performance will depend on site-specific factors, including local feedstock costs, energy prices, and the labour markets.

The economic feasibility of lemon biorefinery operations critically depends on multiple interdependent factors, including plant capacity, product portfolio composition and pricing, feedstock availability and transportation costs, energy requirements and integration opportunities, and prevailing regulatory and incentive frameworks [129,130].

Process simulation studies employing rigorous chemical engineering models, typically implemented in Aspen Plus, have quantified mass and energy flows across various citrus biorefinery configurations. A representative biorefinery processing 381.6 tonnes per hour of wet orange peel biomass—applicable by analogy to lemon processing—can produce 12.2 tonnes of 99.8% bioethanol and 25.8 tonnes per hour of food-grade pectin, alongside 5.9 tonnes per hour of 97% D-limonene recovered at optimal extraction conditions of 90 °C and 0.68 atm pressure [2]. This configuration achieves a D-limonene recovery efficiency of 99.6% relative to the dry biomass content, indicating near-complete extraction of this valuable terpene before downstream processing of depectinized residues [2].

Based on 2010 estimates [11] (adjusted using US Bureau of Labor Statistics Consumer Price Index, 2010–2025), capital investment requirements for lemon biorefinery installations varied substantially with plant capacity and technological complexity. Process design and economic analyses for citrus waste biorefineries producing ethanol, D-limonene, and biogas via dilute-acid hydrolysis pathways indicate that total capital expenditures range from approximately 2.5 million USD for facilities processing 25,000 tonnes per year of citrus waste to 12 million USD for installations handling 400,000 tonnes per year [11]. When adjusted for inflation to reflect 2025 values, the estimated capital investment would increase from approximately USD 2.5 million to around USD 3.7 million for facilities processing 25,000 tonnes per year, and from USD 12 million to roughly USD 17.8 million for installations handling 400,000 tonnes per year. These figures are based solely on general inflation and do not account for potential cost reductions from technological advancements or changes in equipment pricing, which could significantly affect actual capital requirements under current conditions.

Production cost analyses reveal strong economies of scale, with unit costs declining substantially as plant capacity increases. Economic studies indicate that, for ethanol production from a citrus-waste biorefinery by 2010, the minimum selling price (MSP) decreases from USD 2.55 per litre at 25,000 tonnes per year to USD 0.46 per litre at 400,000 tonnes per year, assuming methane and D-limonene prices remain constant [11]. When adjusted for inflation to 2025 values, these figures would rise to approximately USD 3.80 per litre and USD 0.68 per litre, respectively. However, such estimates do not account for technological improvements or market variations in co-products, which could significantly alter the actual MSP under current conditions.

A biorefinery processing 146,000 tonnes per year of citrus peel waste (450 tonnes per day) produces 27.18 million litres per year of anhydrous bioethanol, together with D-limonene, pectin, arabinose and solid residue, with an annual production cost of USD 316 million and a total plant investment of USD 53 million [16].

These economic considerations demonstrate that large-scale implementations benefit from reduced per-unit fixed costs, improved equipment efficiency, and enhanced bargaining power in consumables procurement.

Transportation costs for citrus waste feedstock significantly influence biorefinery economics. Sensitivity analyses indicate that increasing feedstock transportation costs from USD 10 to 30 per tonne elevates ethanol production costs from USD 0.91 to USD 1.42 per litre for a 100,000-tonne-year facility [11]. This dependency underscores the economic imperative to locate strategic biorefineries near concentrated citrus-processing zones or to establish satellite preprocessing facilities to reduce moisture content and volume before long-distance transport [129].

Product portfolio optimisation significantly influences economic returns. Comparative techno-economic assessments of integrated waste cooking oil soap production incorporating citrus-derived D-limonene demonstrate that on-site D-limonene production from citrus peel waste yields an internal rate of return of 19%, compared to 16% for scenarios purchasing D-limonene as an external additive [131]. Minimum selling prices are comparable between the two scenarios (USD 8.88 per kilogram for on-site production versus USD 8.84 per kilogram for purchased additive), both of which remain below the prevailing market price of USD 9.51 per kilogram, thereby establishing economic viability [131]. The preferential economics of integrated production stem from avoided procurement costs, supply chain simplification, and value capture from waste stream valorisation [131].

Energy integration opportunities substantially enhance biorefinery economic performance. The utilisation of biogas generated from the anaerobic digestion of post-extraction residues for on-site heat and electricity generation through cogeneration systems reduces external energy purchases, a significant operational expense [129]. Process simulation and economic modelling demonstrate that biogas recirculation scenarios generate superior economic outcomes compared to external biogas sales, particularly when on-site energy prices reflect industrial rates and cogeneration systems achieve combined heat and power efficiencies exceeding 75% [118].

Market dynamics for lemon biorefinery products influence economic viability projections. D-limonene markets, driven by demand from the cleaning products, cosmetics, pharmaceuticals, and food flavouring industries, have experienced price volatility ranging from USD 3–15 per kilogram, depending on purity specifications and supply-demand balances [2]. Pectin markets, segmented by degree of esterification and applications in the food, pharmaceutical, and cosmetic sectors, exhibit prices ranging from USD 6–25 per kilogram, with high-methoxyl pectin suitable for jams and jellies commanding lower prices than specialised low-methoxyl pectin for dairy applications [68]. Bioethanol and biomethane prices depend heavily on regional renewable energy policies, carbon pricing mechanisms, and fossil-fuel price benchmarks, thereby introducing regulatory and policy-related risks into economic projections [11].

Sensitivity analyses and risk assessments constitute essential components of comprehensive techno-economic evaluations. Key uncertainty parameters typically examined include feedstock costs and availability, product pricing trajectories, energy costs, capital expenditure overruns, and technology performance metrics such as extraction yields and conversion efficiencies [2]. Monte Carlo simulations incorporating probabilistic distributions for critical variables enable the quantification of project risk profiles and identification of viable operating ranges [129]. Results typically indicate that the economics of lemon biorefineries remain attractive under moderately favourable scenarios but become marginal when multiple adverse conditions coincide, such as low product prices coupled with high feedstock and energy costs [11].

Policy and regulatory frameworks have a substantial influence on the economic viability of lemon biorefineries. Renewable energy subsidies, feed-in tariffs for biogas-derived electricity, carbon taxation schemes, waste disposal levies, and research and development tax credits collectively shape the investment landscape [132]. European Union policies promoting circular economy principles and the utilisation of bio-based products, including the Circular Economy Action Plan and revisions to the Renewable Energy Directive, create favourable conditions for biorefinery development through market-pull mechanisms and regulatory push factors [47]. Conversely, regulatory uncertainties regarding novel food ingredients, animal feed approvals, and certifications for bio-based materials may delay market entry and increase commercialisation risks [132].

These economic and environmental analyses collectively demonstrate that integrated lemon biorefinery systems that implement circular-economy principles—particularly those incorporating anaerobic digestion of final residues with biogas recirculation—can achieve superior environmental performance and economic viability compared with conventional linear processing schemes. However, successful implementation requires careful consideration of scale, product portfolio composition, energy integration strategies, feedstock logistics, and regional policy frameworks to ensure both sustainability and profitability outcomes.

The techno-economic considerations outlined in this subsection demonstrate that the economic viability of biorefineries depends fundamentally on plant scale, product portfolio composition, and optimised feedstock logistics.

Table 3 synthesises these interdependencies by presenting a comparative analysis of critical techno-economic parameters across three plant scales: small regional facilities, medium-scale integrated operations, and large centralised biorefineries.

The tabulated data enables prospective investors and technology developers to assess capital requirements, production costs, and profitability indicators as functions of processing capacity, whilst simultaneously evaluating sensitivity to key economic variables such as transportation costs and minimum viable product pricing thresholds.

### 3.4. Business Models and Value Chain Integration

The commercial viability of lemon biorefinery operations depends not merely on technical feasibility and favourable economics, but fundamentally on the business model adopted and the degree of integration achieved within citrus value chains [4]. Three principal business model archetypes have emerged in commercial citrus biorefinery implementations, each offering distinct advantages and constraints depending upon institutional context, investment capacity, market access, and strategic objectives [56,62,133].

Firstly, vertically integrated models in which juice processing companies develop in-house biorefinery capabilities to valorise their own residues offer maximum control over feedstock quality and supply continuity, enable optimisation of processing schedules to match seasonal fruit availability, and capture full value-chain margins from fruit procurement through final product sales [22]. This approach has been successfully demonstrated by large-scale processors in Spain and Italy, which handle more than 50,000 tonnes of citrus fruit annually, thereby justifying investments in sophisticated multi-product biorefinery infrastructure [26]. However, vertical integration requires substantial capital deployment, diverts management attention from core juice production activities, and exposes processors to market risks in unfamiliar product categories, including biochemicals and advanced materials [1,2,14,108].

Secondly, specialised biorefinery operators who procure citrus residues from multiple juice processors under long-term supply contracts provide focused technical expertise, economies of scale through aggregated feedstock volumes, and dedicated market-development efforts for biorefinery products [10]. This model is attractive in regions characterised by fragmented processing industries, in which numerous small- to medium-scale juice producers individually generate insufficient residue volumes to justify standalone biorefinery investments [134]. Successful implementations in Brazil and Argentina demonstrate that integrated biorefinery approaches can achieve profitability by extracting essential oils, pectin, polyphenolic compounds, and producing biogas, thereby creating shared value whilst offering feedstock procurement opportunities that exceed alternative disposal costs [2,132]. Challenges include feedstock supply reliability during off-seasons, variability in quality across suppliers, the need for flexible processing capabilities, and potential competition for residues as biorefinery concepts proliferate [62,135].

Thirdly, cooperative models wherein multiple processors jointly invest in and operate shared biorefinery facilities combine the feedstock security advantages of vertical integration with the scale economies of specialised operators, whilst distributing capital requirements and technical risks across member organisations [136]. Cooperative structures are particularly appropriate for smallholder producer organisations and regional processor associations, in which individual entities lack the resources to develop independent biorefineries, yet collectively command sufficient residue volumes and financial capacity [137]. However, cooperative governance requires robust institutional frameworks for feedstock allocation, cost sharing, revenue distribution, and strategic decision-making, which can be challenging to establish and maintain, particularly in contexts lacking prior cooperative experience [138].

Beyond organisational structure, successful business models demonstrate several common characteristics, including diversified product portfolios to mitigate market risks, phased implementation strategies beginning with established products before progressively incorporating emerging compounds, close collaboration with end-use industries to ensure product specifications match application requirements, and active intellectual property management to protect proprietary extraction methods and product formulations [139,140]. Value chain integration extends downstream through partnerships with food manufacturers, pharmaceutical companies, cosmetic formulators, and materials producers who incorporate citrus-derived ingredients into final products, and upstream through collaborative relationships with citrus growers who benefit from biorefinery-generated organic fertilisers and potentially command premium prices for specific cultivars yielding superior bioactive profiles [10,44]. Industrial symbiosis arrangements in which biorefinery facilities exchange materials, energy, and services with neighbouring industries further enhance economic viability whilst strengthening regional circular-economy networks [141,142].

## 4. Industrial Implementation: Challenges and Opportunities

The transition from laboratory-scale demonstrations to industrial-scale operations of a lemon biorefinery represents a critical juncture in the valorisation of citrus processing residues. Whilst extensive research has validated the technical feasibility of extracting high-value bioproducts from lemon waste, significant challenges persist in achieving commercial viability and market penetration [62,135]. This section examines the scalability constraints, regulatory frameworks, and market opportunities that will determine the successful implementation of integrated lemon biorefinery systems.

### 4.1. Scalability Issues

The scale-up of lemon biorefinery processes from pilot to industrial operations encounters multifaceted technical, economic, and logistical challenges. Recent techno-economic analyses reveal that whilst laboratory-scale extraction of essential oils, pectin, and bioactive compounds demonstrates promising yields, commercial implementation requires substantial capital investment and process optimisation [2,134]. A comprehensive evaluation of citrus biorefinery scalability, conducted by Jeong et al. [143], identified critical bottlenecks in upstream processing, including inconsistent feedstock quality, seasonal availability, and the energy-intensive nature of conventional extraction methods.

Process intensification represents a paramount challenge in industrial-scale lemon biorefinery operations. The implementation of green extraction technologies—such as microwave-assisted extraction, ultrasound-assisted extraction, and supercritical CO_2_—whilst demonstrating superior environmental profiles and extraction efficiencies at laboratory scale, necessitates significant modifications for commercial deployment [144]. Marchette et al. [132] conducted a rigorous process simulation using Aspen Plus v12.1, revealing that scaling limonene extraction to industrial capacity (5.9 tonnes per hour) requires optimal operating conditions of 90 °C and 0.68 atm to achieve a 99.6% recovery efficiency. However, the capital expenditure for such systems remains substantial, with equipment costs accounting for approximately 57% of total biorefinery capital requirements [145].

Energy consumption constitutes a critical constraint on scalability. LCA of citrus biorefineries demonstrates that solvent recycling and water removal through distillation can account for up to 73% of the total biomass energy content, significantly affecting both operational costs and environmental sustainability [145,146]. Lee et al. [135] emphasise that conventional citrus waste management practices are unsustainable in terms of cost and valorisation, and that biorefining requires substantial fuel volumes to meet total energy demand. Consequently, the development of energy-efficient separation technologies and heat-integration strategies is imperative for commercial viability.

Feedstock variability and supply chain logistics present additional scalability challenges. Citrus processing generates waste in concentrated periods corresponding to harvest seasons, necessitating either large-scale storage infrastructure or flexible processing schedules [62]. The seasonal nature of lemon production leads to fluctuating raw material availability, complicating year-round biorefinery operations. Furthermore, the perishable nature of citrus residues—prone to rapid microbial degradation and quality deterioration—demands efficient collection, transportation, and storage systems to maintain feedstock integrity [76].

Market establishment represents a fundamental barrier to scalability. Lee et al. [135] identify the absence of established markets as a significant limiting factor for citrus waste-based biorefining, noting that industrial-scale biorefinery systems are not feasible without secure demand channels for bioproducts. This observation is corroborated by Ortiz-Sánchez et al. [134]. who demonstrated in 2020 through economic analysis that an orange peel biorefinery is economically feasible only at processing scales exceeding 360 tonnes per year, with profitability critically dependent on securing market access for multiple co-products, including essential oils, pectin, and biogas.

Process integration and product diversification strategies have emerged as essential approaches to overcome scalability constraints. Medina-Herrera et al. [62] advocate cascade biorefinery configurations that sequentially extract high-value products, such as essential oils, pectin, and bioactive compounds, followed by conversion of the residual biomass into biofuels or platform chemicals. Such integrated approaches maximise value recovery whilst distributing capital and operational costs across multiple revenue streams, thereby improving economic feasibility. Recent industrial implementations have demonstrated that the co-production of biofuels, limonene, and pectin from citrus wastes can achieve positive financial returns when optimised process integration is employed [147].

Technological maturity varies significantly across different valorisation pathways. Essential oil extraction and pectin production have been successfully implemented on a commercial scale, with established supply chains and quality standards. In contrast, advanced valorisation routes, including nanocellulose production, bioactive compound extraction, and speciality chemical synthesis, remain predominantly at pilot or demonstration scale, requiring further development before widespread industrial adoption [143]. The transition of these emerging technologies from laboratory to commercial scale necessitates sustained research investment, demonstration projects, and industrial partnerships to address technical uncertainties and validate economic performance.

The multifaceted nature of scale-up challenges in implementing a lemon biorefinery requires systematic identification of technical, economic, and operational constraints, along with viable mitigation strategies.

Table 4 synthesises the principal scalability issues confronting industrial-scale lemon biorefinery operations, categorising challenges according to process requirements, feedstock characteristics, capital constraints, technological maturity, market development, and system integration.

For each challenge category, corresponding solutions derived from recent techno-economic assessments and pilot-scale demonstrations are presented, providing a structured framework for biorefinery developers and investors to anticipate and address implementation barriers. The identified solutions reflect current best practices and emerging technologies that have demonstrated efficacy in comparable biomass valorisation systems, specifically adapted for the unique characteristics of lemon processing residues.

### 4.2. Regulatory Aspects

The regulatory landscape has undergone considerable evolution in recent years, with an increasing emphasis on transparency, sustainability, and rigorous safety assessments [147].

Jurisdiction-specific regulatory frameworks across key markets govern the commercialisation of lemon biorefinery products. In the United States, lemon-derived ingredients often follow the *Generally Recognized as Safe* (GRAS) pathway. While traditional extracts enjoy established GRAS status, the FDA has recently signalled increased scrutiny of highly purified derivatives or nanomaterials, emphasising the need for robust safety dossiers comparable to those for food additive petitions.

In contrast, the European Union adopts a more precautionary approach via the Novel Food Regulation (EU) 2015/2283. Products without a significant history of consumption in the EU before 1997—such as nanocrystalline cellulose or highly enriched flavonoid fractions—require pre-market authorisation from the European Food Safety Authority (EFSA). This process involves rigorous toxicological assessment and can extend time-to-market by 18–36 months compared to the US GRAS notification process. Therefore, biorefinery developers must strategically align their product portfolios with regional regulatory landscapes, potentially prioritising established ingredients (such as pectin and essential oils) for immediate EU revenue while developing novel bioactives for markets with more flexible entry pathways.

Food safety regulations constitute the primary regulatory consideration for lemon biorefinery products intended for human consumption. In the United States, the Food and Drug Administration regulates food additives and ingredients under the GRAS provision, which has been subject to significant scrutiny and proposed reforms in 2025. The traditional self-affirmation pathway, whereby companies could independently determine GRAS status without FDA notification, is being phased out in favour of mandatory notification and safety data submission requirements [148]. This regulatory shift necessitates the submission of comprehensive safety dossiers, including toxicological data, exposure assessments, and manufacturing process specifications, for novel lemon-derived ingredients entering the food supply.

The European Union maintains a more stringent precautionary approach to approving food ingredients. Unlike the US system, the EU requires pre-market authorisation through the European Food Safety Authority for all novel food ingredients and additives (Directive 2025/40). This regulatory framework mandates extensive safety evaluations, including genotoxicity testing, subchronic toxicity studies, and comprehensive risk assessments, before commercial authorisation is granted. Consequently, market-entry timelines for novel lemon biorefinery products in European markets typically extend by 18–24 months relative to the United States, with associated regulatory-compliance costs ranging from EUR 200,000 to EUR 500,000 per ingredient dossier.

Lemon peel-derived essential oils enjoy established regulatory status in key markets. D-limonene, the primary constituent of lemon essential oil, is recognised under 21 CFR Part 582 as a GRAS synthetic flavouring substance in the United States, facilitating its widespread use in food, beverage, and cosmetic applications without extensive additional safety documentation [148,149]. Similarly, lemon essential oil is included in the EU’s positive list of flavouring substances [150], enabling its legal use across member states. However, purity specifications, contamination limits, and labelling requirements must be rigorously adhered to for regulatory compliance.

Pectin extracted from lemon peel has a well-established status as a food additive globally. In the Codex Alimentarius, pectin is designated as INS 440, with specifications defined for both high methoxyl and low methoxyl variants. The Food Chemicals Codex provides comprehensive identity and purity standards for commercial pectin, including requirements for galacturonic acid content, degree of esterification, loss on drying, and limits for heavy metals and microbial contaminants. Compliance with these specifications is essential for international trade and market acceptance.

Novel biorefinery products, particularly nanocellulose derivatives and advanced bioactive compounds, are subject to more complex regulatory pathways. Cellulose nanocrystals and nanofibrils, although derived from generally recognised cellulose sources, are considered novel nanomaterials that require specific safety assessments under both EU and US frameworks. The European Commission’s Recommendation 2011/696/EU on the definition of nanomaterials necessitates the comprehensive characterisation of particle size distribution, surface chemistry, and potential toxicological impacts [151,152]. Recent guidance from regulatory agencies emphasises the need for nano-specific risk assessments, including inhalation toxicity, dermal penetration, and environmental fate studies, before commercial applications in food contact materials or dietary supplements can be authorised.

Environmental regulations significantly impact biorefinery operations and product marketing. LCA methodologies, increasingly mandated by corporate sustainability commitments and regulatory initiatives, evaluate the environmental performance of lemon biorefinery processes across multiple impact categories [146]. The EU’s Corporate Sustainability Reporting Directive requires large enterprises to disclose comprehensive environmental data, including greenhouse gas emissions, water consumption, and waste generation, across their entire supply chains. Biorefinery operations that achieve demonstrable reductions in global warming potential, acidification, and eutrophication—as evidenced by rigorous LCA studies—can leverage these environmental credentials for preferential market access and sustainability-focused procurement contracts [68].

Quality certification schemes provide competitive advantages in international markets. Organic certification for lemon biorefinery products requires adherence to strict protocols governing feedstock sourcing, processing methods, and preventing contamination. The USDA National Organic Program and EU Organic Regulation (Regulation EC No. 834/2007) only permit approved extraction solvents and processing aids, with synthetic chemical treatments generally prohibited. Consequently, biorefinery operations targeting organic market segments must implement green extraction technologies and natural processing methods, which may entail higher production costs but can command premium prices in market segments where consumers demonstrate a genuine willingness to pay for sustainability attributes. However, it should be noted that consumer willingness to pay premiums for ‘green’ or ‘eco-friendly’ products is not universal and varies substantially across product categories, geographic markets, income levels, and the credibility of sustainability claims. Empirical evidence suggests that actual purchasing behaviour often falls short of stated preferences, particularly when price differentials exceed 15–20% relative to conventional alternatives [36,46].

Geographical indications and protected designations of origin represent additional regulatory considerations for lemon biorefinery products. European Protected Designation of Origin certifications—such as Limone di Siracusa IGP in Italy or Citron de Menton in France—confer legal protection and market differentiation based on geographical provenance and traditional production methods. Biorefinery operations utilising PDO-certified lemon feedstocks can leverage these designations to enhance market positioning, provided that processing methods comply with established specifications.

International trade regulations influence market access for products from the lemon biorefinery. Tariff classifications under the Harmonised System, phytosanitary certificates for plant-derived products, and compliance with the importing country’s regulations are essential requirements for export operations. The implementation of sustainability-linked trade provisions, including carbon border adjustment mechanisms in the European Union, will increasingly reward low-carbon biorefinery processes with preferential market access.

The complexity of regulatory compliance for lemon biorefinery products varies substantially across jurisdictions and product categories, necessitating a comprehensive understanding of approval pathways, documentation requirements, and timeline expectations.

Table 5 provides a comparative analysis of regulatory frameworks governing the principal product categories from lemon biorefinery operations in the United States and the European Union, which represent the two most extensive and stringent regulatory regimes for food-grade and pharmaceutical ingredients globally. The table delineates specific regulatory classifications, applicable legislation, key specification requirements, and typical approval timelines for each product category.

This comparative framework enables biorefinery operators to develop appropriate regulatory strategies, allocate sufficient resources to compliance activities, and establish realistic market-entry schedules. Notably, the substantial divergence in regulatory philosophies between the United States’ system, which historically permitted greater industry self-determination, and the European Union’s precautionary approach, requiring pre-market authorisation, has significant implications for product development timelines and commercialisation costs.

### 4.3. Market Potential for Each Product

The global market for lemon biorefinery products is experiencing robust growth, driven by rising consumer demand for natural ingredients, stringent sustainability requirements, and expanding industrial applications. Comprehensive market analyses reveal substantial commercial opportunities across multiple product categories, with aggregate market values projected to exceed USD 10 billion by 2035 [153].

Essential oils and limonene represent the most commercially mature product category from lemon biorefinery operations. The global limonene market, valued at USD 351 million in 2024, is projected to reach USD 559 million by 2032, exhibiting a compound annual growth rate of 6.0% [154]. Lemon-derived D-limonene commands a premium price due to its superior organoleptic properties and higher enantiomeric purity compared to orange-sourced variants. The food and beverage sector accounts for approximately 52% of limonene demand, with applications in beverages, confectionery, and baked goods driving market growth [153]. Cosmetics and personal care applications are experiencing the most rapid expansion, with a 7.37% CAGR, driven by consumer preferences for natural fragrances and bio-based solvents in skincare formulations [155]. Industrial cleaning applications maintain a stable demand, with limonene’s efficacy as a natural degreaser and its favourable environmental profile positioning it as an attractive alternative to petroleum-based solvents.

Lemon essential oil, which comprises 55–70% limonene and minor constituents such as β-pinene, γ-terpinene, and citral, occupies a distinct market segment. The global *Citrus* × *limon* peel oil market is estimated at USD 450 million in 2024, with projections indicating growth to USD 600 million by 2029, at a 5% CAGR [155]. Cold-pressed lemon oil commands premium prices ranging from USD 15–25 per kilogram for food-grade specifications, whilst pharmaceutical-grade variants exceed USD 40 per kilogram [10,156]. The aromatherapy and wellness sectors have emerged as high-growth areas, with lemon oil positioned as a leading product in the citrus essential oil category due to its documented anxiolytic properties, antimicrobial activity, and cognitive-enhancing effects.

Pectin extracted from lemon peel represents a substantial market opportunity within the hydrocolloid sector. The global citrus pectin market, valued at USD 1.3 billion in 2024, is forecast to expand at a CAGR of 5.5–7% through 2034, reaching approximately USD 2.1 billion [157]. High-methoxyl pectin, which accounts for approximately 63% of the market share, is primarily used in jams, jellies, and fruit preparations, where its gelling properties under high-sugar conditions are essential [158,159,160]. Low-methoxyl pectin, although a smaller market segment, is experiencing accelerated growth at a 7.4% CAGR, driven by demand for reduced-sugar and vegan food formulations [161]. Pharmaceutical applications of modified citrus pectin—particularly for chelation therapy and as a component in controlled-release drug delivery systems—represent emerging high-value opportunities with selling prices exceeding USD 20,000 per tonne for pharmaceutical-grade material.

Pricing dynamics in the pectin market reflect supply and demand imbalances and raw-material availability. Recent data indicate average pectin prices of USD 10,823–11,385 per tonne in significant markets, with variations attributable to feedstock costs, production technology, and quality specifications [157]. The 24% decline in Brazilian citrus harvests from 2024 to 2025, due to citrus greening disease, has exerted upward pressure on pectin prices, encouraging diversification into alternative sources, including lemon peel [155]. Lemon-derived pectin, characterised by its low methoxyl content and excellent gelation properties, can command premium pricing in specialised applications.

Bioactive compounds extracted from lemon residues—including flavonoids, phenolic acids, and dietary fibres—constitute an emerging high-value product category. Hesperidin, a flavanone glycoside abundant in citrus peel, commands market prices ranging from USD 15 to 30 per kilogram for food-grade material, with pharmaceutical-grade material exceeding USD 80 per kilogram. The global citrus bioflavonoids market, encompassing hesperidin, naringin, and related compounds, is experiencing robust growth driven by rising demand for nutraceutical and functional food applications. Eriocitrin, another prominent flavonoid in lemon peel, demonstrates potent antioxidant and anti-inflammatory properties, positioning it favourably for dietary supplement formulations. Market analysts project an increasing demand for lemon-derived bioactive compounds as scientific evidence supporting their health benefits accumulates and consumer awareness of functional ingredients expands.

Nanocellulose products derived from lemon peel residues offer ultra-high-value market opportunities, though they are less commercially mature than essential oils and pectin. The global nanocellulose market, valued at USD 490–580 million in 2024–2025, is projected to reach USD 2.26–3.16 billion by 2033–2034, with a projected CAGR of 18.5–20.1% [162,163]. Cellulose nanocrystals command premium prices of USD 150–300 per kilogram for research-grade material. In contrast, commercial-scale production is projected to reduce costs to USD 5–15 per kilogram, facilitating broader market penetration. Applications in packaging, composites, and biomedical materials drive demand, as cellulose nanocrystals are valued for their exceptional mechanical properties, biodegradability, and barrier properties. Lemon peel-derived α-cellulose, following chemical purification, serves as an excellent feedstock for nanocellulose production, potentially generating a value of USD 1500–3000 per tonne of dry lemon peel processed. Realising these market projections, however, requires demonstration projects that validate integrated multi-product processing at pilot and industrial scales; recent pilot-scale citrus biorefinery studies have confirmed the technical feasibility of sequential extraction schemes but underscored the need for further scale-up optimisation and technology readiness advancement before full commercial deployment [164,165].

Citric acid, whilst conventionally produced through fermentation, can be recovered from lemon processing streams, particularly juice fractions. The global citric acid market, valued at approximately USD 3.5 billion in 2024, is projected to grow steadily at a 4–5% CAGR [166,167]. Food-grade citric acid prices range from USD 800–1200 per tonne, whereas anhydrous pharmaceutical-grade citric acid commands USD 1500–2000 per tonne. While fermentation-based production dominates the commercial supply, integrating citric acid recovery into lemon biorefinery configurations can provide additional revenue streams, particularly for operations with co-located juice-processing facilities.

Bioenergy products—including bioethanol, biogas, and solid biofuels—derived from residual lemon biomass following high-value compound extraction represent baseline valorisation options. Bioethanol produced from fermentable sugars in lemon peel achieves market values of approximately USD 0.66–0.75 per litre of gasoline-equivalent, whilst biogas generated through anaerobic digestion of post-extraction residues provides renewable energy for biorefinery operations or for sale to energy markets. Although bioenergy products yield lower per-tonne revenues than biochemicals, their role in achieving zero-waste biorefinery configurations and improving overall process economics remains significant.

The commercial attractiveness of lemon biorefinery operations fundamentally depends on the market values, growth trajectories, and demands for constituent bioproducts derived from cascade valorisation processes. Translating these market opportunities into viable commercial ventures will additionally require coordinated market development initiatives, encompassing standardisation of product specifications, certification pathways for novel bio-based products, and supportive policy frameworks. International strategies such as the FAO Bioeconomy Strategy and the EU Circular Economy Action Plan provide enabling conditions for market creation by promoting bio-based value chains, harmonising quality standards, and incentivising the substitution of fossil-derived products with renewable alternatives [168,169].

Table 6 presents comprehensive techno-economic indicators available from integrated lemon biorefinery systems, including current market valuations, projected growth through 2030–2035, compound annual growth rates (CAGRs), primary application sectors, and key factors driving market expansion.

The data synthesised in this table are derived from authoritative market research publications and industry analyses conducted during 2024–2025, ensuring the projections’ currency and reliability. The substantial variation in market sizes, growth rates, and value propositions across product categories underscores the strategic importance of portfolio optimisation in biorefinery design. Products spanning mature commodity markets with moderate growth rates, such as pectin and limonene, provide revenue stability and established distribution channels, whilst emerging high-value products, including nanocellulose and bioactive compounds, offer exceptional growth potential, albeit with greater market development requirements. This market intelligence serves as the foundation for strategic decision-making regarding product prioritisation, capacity allocation, and market-entry sequencing in commercial biorefinery implementations.

Integrated techno-economic analyses demonstrate that diversified product portfolios maximise economic returns from lemon biorefinery operations. Marchette et al. [132] reported that a biorefinery processing 381.6 tonnes per hour of wet orange biomass can produce 12.2 tonnes of 99.8% ethanol, 25.8 tonnes per hour of pectin, and 5.9 tonnes per hour of limonene (97% purity), achieving favourable economic indicators. Extending this framework to a lemon biorefinery that sequentially extracts essential oils, pectin, bioactive compounds, nanocellulose, and bioenergy from residual biomass can generate aggregate revenues of USD 400–600 per tonne of dry lemon peel processed, assuming optimised extraction efficiencies and established market channels.

Market penetration strategies require careful attention to product positioning, quality differentiation, and sustainability credentials. The organic and natural product segments command premium prices, with organic-certified lemon-derived ingredients commanding 20–40% price premiums over conventional variants. Sustainability certifications, including verification of carbon neutrality and compliance with circular-economy principles, are increasingly influencing purchasing decisions in corporate supply chains. LCA documentation demonstrating reduced environmental impacts compared to conventional products enhances marketability, particularly in European markets where sustainability reporting requirements are stringent [68].

Regional market dynamics present both opportunities and challenges for lemon biorefinery products. North America accounts for approximately 32–35% of global demand for limonene and pectin [153,168]. However, the Asia–Pacific region exhibits the highest growth rates, driven by the expansion of food-processing industries, rising disposable incomes, and increasing consumer demand for natural ingredients in personal care products. Europe maintains a strong demand for high-quality pectin and essential oils, with stringent regulatory standards that favour established suppliers with comprehensive quality documentation.

Competitive positioning within fragmented markets requires strategic differentiation. Major players in the pectin market, including CP Kelco, Cargill, DuPont, and Herbstreith & Fox, maintain competitive advantages through vertical integration, proprietary extraction technologies, and established customer relationships. New entrants, including lemon biorefinery operations, can compete effectively through specialised product offerings (e.g., low-methoxyl pectin, organic-certified ingredients), regional supply-chain advantages, and superior sustainability profiles. The nanocellulose market, characterised by limited commercial-scale producers and high barriers to entry, offers first-mover advantages in specific application segments.

Price volatility in feedstock and product markets necessitates robust risk management strategies. Seasonal fluctuations in the availability of lemon processing residue, variability in essential oil yields due to cultivar and growing conditions, and global supply and demand dynamics for pectin influence profitability. Long-term supply agreements with citrus processors, flexible production scheduling to accommodate seasonal variations, and hedging strategies for key inputs and outputs can mitigate market risks.

The trajectory towards commercial success in lemon biorefinery operations ultimately depends on achieving competitive cost structures whilst delivering superior product quality and sustainability performance. As extraction technologies mature, economies of scale are realised, and market awareness of lemon-derived bioproducts expands, the commercial viability of integrated lemon biorefinery systems will strengthen substantially, positioning this valorisation approach as a cornerstone of the circular bioeconomy in the citrus processing sector.

The economic viability and environmental sustainability of integrated lemon biorefinery systems ultimately depend on achieving performance benchmarks across technical, financial, and ecological dimensions.

Table 7 consolidates the market values and growth projections derived from recent process simulations, life-cycle assessments, and pilot-scale demonstrations of citrus biorefinery operations, providing quantitative targets and operational parameters essential to commercial success.

These indicators include minimum viable processing scales, capital and operating expenditure requirements, revenue-generating potential, energy-efficiency metrics, environmental impact assessments, and financial performance measures, such as payback periods and Internal Rates of Return (IRR). The ranges presented reflect variations in biorefinery configurations, technological choices, feedstock characteristics, and market conditions documented in peer-reviewed literature and industry reports. Attention is focused on identifying optimal operating conditions and critical success factors that differentiate economically viable operations from marginal or unprofitable ones. This performance framework serves as a benchmarking tool for biorefinery developers conducting feasibility assessments, enabling comparison of proposed designs with demonstrated best practices and facilitating the identification of opportunities for improvement. Achieving indicator values within the specified optimal ranges is a prerequisite for attracting investment capital, securing project financing, and establishing commercially sustainable lemon biorefinery operations that contribute meaningfully to circular economic objectives while generating acceptable financial returns for stakeholders.

### 4.4. Market Development and Demand Analysis

The commercial success of lemon biorefinery operations ultimately depends on robust, stable markets for recovered products at prices sufficient to justify processing investments and generate attractive financial returns [143]. Market development for citrus-derived products faces distinct challenges across product categories, application sectors, and geographic regions, necessitating strategic approaches tailored to specific contexts [8].

Products such as essential oils, pectin, and seed oils benefit from mature markets with well-defined quality standards, established distribution channels, and predictable pricing dynamics, thereby facilitating straightforward market integration for citrus-derived variants, provided they meet industry specifications [172]. Global markets for citrus essential oils were valued at approximately USD 4.2 billion in 2023, growing at a compound annual growth rate (CAGR) of roughly 4.5%, driven by demand for natural fragrances in personal care products and clean-label flavourings in processed foods [173]. Pectin markets similarly demonstrate steady growth, with global consumption valued at over USD 1.1 billion in 2023 and projected to reach USD 2.1 billion by 2030, driven by its use as a natural gelling agent in jams, jellies, confectionery, and pharmaceutical formulations, with citrus peels accounting for approximately 85.5 per cent of commercial pectin production [174].

Emerging products, including polyphenolic extracts, industrial enzymes, and nanocrystalline cellulose, face substantially greater market development challenges, as potential customers often lack familiarity with product properties, regulatory approval pathways remain under development, and price discovery proves difficult due to limited commercial availability [61]. Citrus-derived hesperidin and naringin extracts compete with synthetic alternatives and orange-derived products in nutraceutical markets, requiring demonstration of superior bioavailability or enhanced bioactivity to justify price premiums [175]. Industrial enzyme markets, whilst substantial in aggregate, exceeding USD 7 billion globally, demonstrate strong incumbent positions held by established producers, who offer comprehensive product catalogues and technical support services that new entrants struggle to match [176]. Nanocrystalline cellulose markets remain nascent despite extensive research interest, with a global market size estimated at approximately USD 500 million in 2024 and prices ranging from USD 5 to 200 per kilogram, depending on purity and functionalisation, thereby constraining applications to high-value speciality products rather than commodity materials [177].

Consumer willingness to pay (WTP) a premium for products derived from environmentally responsible biorefinery processes represents a decisive yet frequently underestimated variable in market viability assessments. Meta-analyses of WTP studies across food ingredients, nutraceuticals, and sustainable materials markets indicate that green product premiums of 10–30% are achievable among health-conscious and environmentally motivated consumer segments, particularly when sustainability credentials are communicated transparently through credible certification schemes, traceability systems, or independent third-party endorsements [48,49,178]. However, premium WTP erodes substantially in price-sensitive commodity markets and when consumers perceive no meaningful functional differentiation between biorefinery-derived and conventionally sourced products.

For lemon biorefinery outputs, this dynamic implies a differentiated market positioning strategy: high-value fractions—including pharmaceutical-grade pectin, standardised polyphenolic extracts with demonstrated bioactivity, and high-purity nanocrystalline cellulose—may sustain viable green premiums within niche markets where performance specifications, provenance traceability, and circular-economy credentials carry genuine commercial weight.

Conversely, commodity-grade essential oils and feed-grade biomass fractions face strong downward pricing pressure, constraining the commercially realisable premium. Translating sustainability value into consumer price acceptance, therefore, requires deliberate market segmentation, investment in green brand equity, and strategic co-development partnerships with buyers who embed circular-economy narratives into their own product positioning.

Demand analysis for citrus biorefinery products must consider multiple factors, including market size and growth rates; competitive positioning relative to alternative sources; regulatory requirements that influence market access; customer willingness to pay for sustainable or natural-origin products; and the potential for market creation through novel applications not available with conventional materials [179]. Consumer preference trends strongly favour natural-origin ingredients over synthetic alternatives in food, cosmetic, and pharmaceutical applications, with systematic reviews indicating that consumers are willing to pay price premiums of approximately 5 to 30% for healthier or natural food products compared to synthetic equivalents, provided regulatory approval and consistent quality can be assured [49]. Industrial customers are increasingly interested in bio-based materials as part of corporate sustainability commitments and supply chain decarbonisation strategies, creating opportunities for citrus-derived cellulose and platform chemicals to displace petroleum-based incumbents despite their initially higher costs [180]. Public procurement policies in European Union member states and other jurisdictions increasingly mandate consideration of environmental performance and circular-economy contributions in purchasing decisions, potentially accelerating the adoption of bio-based products through guaranteed initial demand volumes [181].

Strategic market development approaches combine direct customer engagement to understand application requirements and co-develop product specifications, participation in industry trade associations and standard-setting bodies to influence market norms favourably, collaboration with research institutions to generate application data demonstrating product performance, and selective geographic focus on regions combining supportive regulatory environments with strong sustainability orientation and willingness to trial novel ingredients. Early market entry in high-value niche applications generates revenue whilst building technical expertise and customer relationships, enabling expansion into larger-volume markets as production scales and costs decline [62].

### 4.5. Scale-Up Challenges and Technology Transfer

The translation of laboratory-scale extraction advances and pilot-scale biorefinery demonstrations into commercially viable, reliably operating industrial facilities faces numerous technical, economic, and institutional challenges that collectively constitute the “valley of death,” which claims most promising technologies before they achieve commercial deployment [182]. Scale-up challenges span multiple dimensions, including equipment design and fabrication, process control and automation, feedstock variability management, product quality consistency, regulatory compliance demonstration, workforce training, and organisational capacity building, and require systematic attention throughout the technology development and commercialisation phases [7].

Equipment scaling from laboratory glassware and pilot-plant units to industrial-scale reactors, extractors, and separators presents non-trivial engineering challenges, including mixing efficiency, heat-transfer rates, mass-transfer kinetics, and residence-time distributions, all of which exhibit scale dependencies that simple geometric scaling relationships cannot reliably predict [62]. Ultrasound-assisted extraction (UAE) systems exemplify these challenges: acoustic intensity, cavitation bubble dynamics, and tissue-disruption efficiency vary substantially with vessel geometry, transducer configuration, and processing volumes, necessitating careful pilot-scale validation before committing to full commercial implementation [115]. Recent pilot-scale studies demonstrate that UAE parameters optimised at a laboratory scale (e.g., 49.92 W·cm^−2^ intensity) require substantial recalibration for flow-through systems processing hundreds of kilograms of citrus peel waste per hour [115]. Microwave-assisted extraction faces similar complexities regarding electromagnetic-field uniformity, temperature-distribution homogeneity, and thermal-runaway prevention in large-volume systems [183]. Supercritical fluid extraction systems incur elevated capital costs at commercial scale due to high-pressure equipment requirements, complex instrumentation, and safety systems mandated by process hazards, with energy-intensive operations imposing additional economic constraints [29]. Enzymatic extraction systems must address enzyme recovery and recycling to manage costs whilst maintaining product quality, requiring robust downstream processing, including enzyme inactivation, ultrafiltration, and, where appropriate, enzyme immobilisation on solid supports to enable continuous-flow operation [5].

Beyond these technical scale-up hurdles, the economic feasibility of green extraction technologies at an industrial scale remains a central question for biorefinery commercialisation. Whilst UAE, MAE, pulsed electric field (PEF), supercritical fluid extraction (SFE), and enzymatic processes consistently demonstrate superior selectivity, reduced solvent consumption, and lower environmental footprints at laboratory and pilot scales, their economic competitiveness relative to conventional solvent extraction varies substantially by technology and processing context [29,115,183]. UAE and MAE systems are approaching economic parity with conventional extraction at processing volumes exceeding 10,000 tonnes per year, as declining equipment costs, improved energy efficiency of modern transducer and magnetron designs, and reduced solvent procurement and disposal expenditures progressively narrow the operating cost differential [115]. SFE using supercritical CO_2_ remains significantly more capital-intensive, with capital expenditure requirements typically 3–5 times higher than those of conventional extraction systems of equivalent throughput, owing to the high-pressure vessels, precision pumping systems, and associated safety infrastructure required [29]. Nevertheless, SFE offers compelling advantages for high-value, solvent-free product streams, particularly pharmaceutical-grade essential oils and nutraceutical extracts—where the premium pricing commanded by solvent-free certification can offset the higher processing costs. Enzymatic extraction becomes economically viable when enzyme recycling through ultrafiltration membrane systems or enzyme immobilisation on solid supports is implemented, reducing the cost contribution of commercial enzyme preparations from 15–25% of total operating expenditure to less than 5%, thereby rendering the technology competitive with acid- or alkali-based alternatives for pectin and bioactive compound recovery [5]. Taken together, the current evidence suggests that a hybrid approach—combining conventional mechanical or thermal pre-treatment steps with green extraction technologies for polishing or targeted compound recovery—offers the most favourable balance of economic feasibility and product quality at commercial scales. Full adoption of exclusively green processing chains is expected to become increasingly viable as equipment costs decline through technological maturation, manufacturing scale effects, and the growing availability of standardised modular extraction units designed for the food and biorefinery industries [7,115].

Process control and automation systems essential for commercial operation must integrate multiple unit operations, including feedstock reception and preparation, extraction reactors, solid–liquid separation systems, solvent recovery and recycling, product purification, and final packaging, whilst maintaining product quality specifications, maximising yields, minimising energy consumption, and ensuring operator safety [3]. Modern biorefinery facilities increasingly adopt Industry 4.0 concepts, including real-time process monitoring through distributed sensor networks, automated quality control using inline analytical instruments, predictive maintenance enabled by machine-learning algorithms that analyse equipment performance data, and digital-twin simulation models that support operator training and process optimisation [44]. However, technology transfer to developing regions and small-scale processors is often constrained by limited access to sophisticated control systems, insufficient technical expertise for system programming and maintenance, and cost barriers to advanced instrumentation [184].

Feedstock variability management is a particularly acute challenge for citrus biorefineries, as residue composition varies substantially across citrus varieties, fruit maturity, seasonal factors, growing regions, harvest years, and juice-extraction methods. Pectin content in lemon peels ranges from 20 to 30% on a dry-weight basis, depending on the factors [185], whilst essential oil content ranges from 0.8 to 2.55% and polyphenol concentrations span 2.1 to 6.2 mg gallic acid equivalents per gram of dry peel. Such variability directly affects extraction yields, product purity, and processing costs, necessitating either accepting variable product specifications that are unsuitable for demanding applications or implementing adaptive processing protocols that adjust extraction parameters based on real-time feedstock characterisation [186]. Advanced approaches employ rapid analytical methods, including near-infrared spectroscopy for feedstock characterisation, enabling immediate process adjustments, whilst alternative strategies establish feedstock blending protocols combining residues from multiple sources to achieve a consistent average composition [5].

Product quality consistency proves critical for market acceptance, particularly in pharmaceutical and food applications, where regulatory requirements mandate tight specifications and batch-to-batch reproducibility [5]. Achieving consistent quality across variable feedstock processes in complex multi-stage biorefinery operations requires comprehensive quality management systems that encompass incoming feedstock inspection, in-process monitoring at critical control points, final product testing against established specifications, and statistical process control methodologies to identify trends that require corrective action before specifications are breached [187]. Technology transfer initiatives must emphasise the development of quality management systems alongside equipment installation and process training to ensure commercial viability [188].

Regulatory compliance demonstrations for novel products require extensive documentation, including process descriptions, quality control protocols, analytical method validation data, stability studies, toxicological assessments, and, for bioactive compounds intended for pharmaceutical applications, potentially clinical trials [5]. The European Food Safety Authority (EFSA) has issued specific guidance on the risk assessment of nanomaterials and novel foods. Yet implementation pathways for agro-waste-derived products remain fragmented, creating barriers to market entry [189]. Small-scale processors and developing-region enterprises often lack the resources and expertise to prepare comprehensive regulatory dossiers, suggesting opportunities for industry associations or government agencies to develop shared technical resources, coordinate multi-company testing programmes, and provide regulatory guidance tailored to citrus biorefinery products [190]. Successful technology transfer ultimately depends on holistic approaches that address not merely equipment and process knowledge but also institutional capacity building, workforce training, supply chain development, and the establishment of market linkages [73].

## 5. Future Perspectives and Research Needs

Building upon the strategic roadmap delineated in Part I. The transformation of lemon processing residues into high-value bioproducts through cascade biorefinery approaches has demonstrated substantial technical feasibility and promising economic potential. However, the transition from laboratory-scale demonstrations to commercially viable industrial operations necessitates addressing critical knowledge gaps, technological challenges, and systemic barriers that currently constrain widespread implementation. This section delineates priority research directions, emerging technological frontiers, and strategic imperatives that will shape the future trajectory of lemon waste valorisation within the circular bioeconomy framework.

### 5.1. Advanced Process Integration

The optimisation of cascade biorefinery configurations represents a paramount research priority, requiring systematic investigation of sequential extraction pathways, process synergies, and resource integration strategies. Whilst individual valorisation routes—such as essential oil extraction, pectin recovery, and bioactive compound isolation—have been extensively characterised, comprehensive studies examining the interdependencies, trade-offs, and cumulative effects of integrated multi-product systems remain notably scarce [191]. Future research should employ advanced process simulation methodologies, including computational fluid dynamics, artificial neural networks, and multi-objective optimisation algorithms, to identify optimal cascade sequences that maximise total value recovery whilst minimising energy consumption, solvent usage, and environmental impacts [192].

The development of predictive models that can accommodate feedstock variability represents a critical research need. The composition of lemon peel varies substantially with cultivar, maturity stage, growing conditions, and post-harvest handling, directly influencing extraction yields and product quality [193]. Machine learning approaches employing spectroscopic fingerprinting techniques—such as near-infrared spectroscopy, Raman spectroscopy, and hyperspectral imaging—coupled with chemometric analysis offer promising avenues for real-time feedstock characterisation and adaptive process control [194,195]. Recent advances in Industry 4.0 technologies, including Internet of Things sensors, digital twins, and cloud-based analytics platforms, enable continuous monitoring and dynamic optimisation of biorefinery operations, ensuring consistent product quality despite inherent feedstock heterogeneity [196].

Energy integration strategies warrant greater research attention, given that energy consumption is a major operational expense and environmental burden in citrus biorefinery systems. Pinch analysis methodologies, combined with exergy analysis, should be systematically applied to identify optimal heat exchanger networks, minimise irreversibilities, and maximise energy recovery opportunities. The integration of renewable energy systems—solar thermal collectors, photovoltaic arrays, biomass boilers utilising residual solid fractions—with biorefinery operations offers pathways to energy self-sufficiency and carbon-neutral production, meriting comprehensive techno-economic and LCAs [197].

### 5.2. Regulatory Frameworks for Citrus-Derived Products

The commercial deployment of lemon biorefinery products confronts complex, evolving regulatory landscapes that vary substantially across jurisdictions, product categories, and intended applications, requiring careful navigation to achieve market access whilst ensuring consumer safety and environmental protection [29]. Regulatory frameworks governing citrus-derived products span multiple domains, including food safety, novel food approval, pharmaceutical regulation, cosmetic ingredient registration, nanomaterial classification, and environmental compliance, each administered by distinct agencies employing different assessment criteria, approval processes, and timelines [198]. This regulatory complexity is particularly challenging for integrated biorefineries that produce diverse product portfolios for multiple application sectors, as each product stream may face distinct regulatory requirements [199]. These necessitate parallel approval efforts that consume substantial resources and extend commercialisation timelines [29].

#### 5.2.1. Food and Beverage Applications

Citrus-derived ingredients intended for food and beverage applications are subject to regulatory oversight primarily through food safety and novel food frameworks, with specific requirements varying substantially across major markets, including the European Union, the United States, and emerging economies [200]. Within the European Union, the Novel Food Regulation (EU) 2015/2283 governs foods and food ingredients that were not consumed to a significant degree within the Union before 15 May 1997, requiring pre-market safety assessment and explicit authorisation before commercialisation [201]. This framework applies to numerous citrus biorefinery products, including nanocrystalline cellulose (due to engineered nanomaterial status), highly purified polyphenolic extracts exceeding historical consumption levels, and novel derivatives of pectin or cellulose created through chemical modification [30,198]. The application process requires comprehensive dossiers documenting product composition, proposed uses and use levels, anticipated intake estimates, nutritional impact assessments, and toxicological evaluations, including genotoxicity, subchronic toxicity, and allergenicity studies where appropriate [30]. Recent analysis of 292 novel food applications submitted to EFSA between 2018 and 2024 revealed an average duration from application submission to opinion publication of approximately 2.6 years, with substantial variability and delays primarily attributable to suitability checks and additional data requests; some applications took up to 6 years [31].

In contrast, citrus-derived products with an established history of food use before 1997 generally qualify for approval under traditional food frameworks that require demonstrating safety through existing consumption patterns rather than extensive toxicological testing [200,202]. Essential oils, pectin, and citrus extracts containing flavonoids at concentrations consistent with traditional preparation methods are typically not considered novel foods, thereby substantially reducing regulatory barriers and enabling faster market entry [203,204]. However, concentration or purification processes that yield products with bioactive compound levels significantly exceeding those in traditional preparations may trigger novel food assessment requirements, creating regulatory uncertainty and necessitating careful consultation with competent national authorities before commercialisation [30]. The European Food Safety Authority guides this process through its Panel on Nutrition, Novel Foods and Food Allergens, though precedents for specific citrus-derived products remain limited, necessitating case-by-case evaluations [30]. In the context of agro-food by-product valorisation, EFSA published 45 scientific opinions on the safety of novel foods, food enzymes, and food additives derived from plant and animal by-products through December 2023, reflecting growing interest in circular-economy applications [198].

United States regulation occurs primarily through the Food and Drug Administration’s GRAS framework or food additive petition processes [205]. Substances with established safety histories based on scientific procedures or on food use before 1958 may qualify for GRAS status through either self-determination by qualified experts or a formal GRAS notice submitted to the FDA. Citrus peel extracts, essential oils, and pectin preparations have established GRAS status for traditional uses, enabling straightforward market access, provided manufacturing processes and purity specifications align with historical practices. Citrus fibre, for example, has received GRAS status for use as a texturiser and moisture-retention agent in various food applications. Novel derivatives, highly purified fractions, or products at significantly elevated concentrations relative to conventional uses require either demonstration of GRAS status through comprehensive safety assessments conducted by independent expert panels or submission of food additive petitions that provide extensive toxicological data. The FDA increasingly scrutinises GRAS determinations for novel ingredients, particularly those involving nanotechnology or significant purification beyond traditional food forms, potentially requiring data packages that approach the comprehensiveness of a food additive petition, despite nominally streamlined GRAS pathways [205]. Unlike the centralised EU system, in which EFSA conducts scientific evaluations and the European Commission makes authorisation decisions, the US offers multiple regulatory pathways depending on the nature of the novel food, with the GRAS process generally allowing faster market access but placing greater responsibility on companies to ensure product safety [206].

#### 5.2.2. Pharmaceutical and Nutraceutical Applications

Citrus-derived bioactive compounds intended for pharmaceutical applications face substantially more rigorous regulatory oversight than food ingredients, requiring the demonstration of safety and efficacy through clinical trial programmes and comprehensive quality control systems that conform to Good Manufacturing Practice standards [207]. Within the European Union, pharmaceutical products must obtain Marketing Authorisation from the European Medicines Agency or national competent authorities following evaluation of preclinical safety studies, clinical pharmacokinetic data, efficacy demonstrated through controlled clinical trials, and manufacturing quality systems that ensure consistent product specifications [208]. This approval pathway is prohibitively expensive and time-consuming for most citrus biorefinery products, with recent analyses indicating that drug development costs range from USD 172.7 million (out-of-pocket expenses) to USD 879.3 million when accounting for failures and capital costs, whilst timelines typically span 10 to 15 years from initial development through regulatory approval [209,210].

Consequently, most citrus-derived bioactive products target the nutraceutical and dietary supplement markets, which operate under less stringent regulatory frameworks that permit health-related marketing claims without requiring pharmaceutical-level efficacy demonstration, provided certain conditions are met [200]. European Union regulations distinguish between medicinal products requiring Marketing Authorisation and food supplements governed by food law, with classification depending upon product presentation, dosage form, bioactive concentrations, and claimed effects. Products making disease treatment or prevention claims generally require pharmaceutical approval, whilst those making structure-function claims (supporting normal physiological functions) or reduction-of-disease-risk claims may proceed under food supplement frameworks, provided the claimed effects are substantiated by credible scientific evidence [211]. The European Food Safety Authority’s Panel on Nutrition, Novel Foods and Food Allergens evaluates health claim applications under Regulation (EC) No 1924/2006, which requires demonstrating cause-and-effect relationships between consumption and claimed benefits through intervention studies, preferably conducted in human subjects [212]. The distinction between medicinal products and food supplements remains a significant regulatory challenge, with borderline classification issues creating uncertainty for manufacturers of bioactive-rich citrus extracts [213].

United States regulation of dietary supplements is governed by the Dietary Supplement Health and Education Act (DSHEA) of 1994, which requires manufacturers to ensure product safety without premarket approval but permits structure-function claims, provided they are truthful, not misleading, and substantiated by credible scientific evidence [214]. New dietary ingredients not marketed in the United States before 15 October 1994 require pre-market notification to the FDA, including safety evidence; however, approval is not explicitly required, and FDA objections are relatively uncommon, absent clear safety concerns [215]. This regulatory approach enables faster market entry for citrus-derived nutraceuticals than for pharmaceuticals or the European Union novel food pathways. However, ongoing calls for regulatory modernisation and recent enforcement actions against products that make insufficiently substantiated claims suggest an increasing regulatory scrutiny of the dietary supplement market [214,216]. The global nutraceutical market continues to expand rapidly, with citrus flavonoids such as hesperidin and naringin demonstrating considerable therapeutic potential for cardiovascular health, management of metabolic syndrome, and anti-inflammatory therapies [84,217].

#### 5.2.3. Cosmetic and Personal Care Applications

Cosmetic ingredients derived from citrus processing waste are subject to less stringent regulatory requirements than those for food or pharmaceutical applications in most jurisdictions, although specific regulations vary substantially. European Union Cosmetic Regulation (EC) No 1223/2009 requires safety assessment by qualified personnel and notification through the Cosmetic Products Notification Portal [218]. Still, it does not mandate premarket approval unless ingredients appear on restricted-substance lists or novel nanomaterial forms are employed, both of which require specific risk assessments [219]. Essential oils, pectin derivatives, and polyphenolic extracts from citrus generally qualify as natural cosmetic ingredients exempt from restricted substance provisions, provided they contain no prohibited substances and meet purity specifications for heavy metals, microbial contamination, and pesticide residues [220]. Nanocrystalline cellulose intended for cosmetic applications requires specific notification that identifies its nanomaterial status and provides risk assessments addressing dermal exposure [220,221]. However, outright prohibitions remain uncommon for low-toxicity cellulose-based nanomaterials [222,223].

United States cosmetic regulation is even less stringent, as the Food, Drug, and Cosmetic Act does not require pre-market approval for cosmetic products or ingredients, except for colour additives [224]. Manufacturers are responsible for ensuring safety but need not submit safety evidence to the FDA before marketing, unless their products make drug claims that require pharmaceutical regulation.

#### 5.2.4. Nanomaterial-Specific Regulations

Nanocrystalline cellulose and other nanoscale materials derived from citrus processing waste are subject to additional regulatory considerations beyond conventional substance frameworks, given concerns about their potential toxicological properties at the nanoscale [225]. The European Union’s definition of nanomaterial encompasses materials wherein fifty per cent or more of particles by number exhibit one or more external dimensions between one and one hundred nanometres [226], capturing nanocrystalline cellulose products typically demonstrating dimensions of five to twenty nanometres in width and one hundred to three hundred nanometres in length [227]. Novel Food Regulation (EU) 2015/2283 explicitly includes engineered nanomaterials within its scope [198], requiring a pre-market safety assessment that addresses nanospecific endpoints, including particle size distribution, surface properties, aggregation behaviour, biokinetics, and potential for translocation across biological barriers [59]. Similarly, cosmetic regulation mandates specific notification and risk assessment for nanomaterials [218], although cellulose-based materials, which demonstrate negligible toxicity and limited dermal penetration, generally receive favourable assessments. United States regulatory approaches to nanomaterials remain less prescriptive, with FDA guidance documents recommending a case-by-case evaluation to determine whether nanoscale dimensions alter safety, efficacy, or quality characteristics compared to conventional-scale materials. The agency declined to establish blanket nanomaterial regulations, instead emphasising consideration of size-dependent effects within existing product-specific frameworks. This approach creates uncertainty for nanocrystalline cellulose producers about whether their products require novel food evaluation or qualify for existing cellulose approvals, necessitating early FDA consultation for market-entry planning [228].

#### 5.2.5. Environmental and Occupational Safety Regulations

Biorefinery facilities processing citrus waste must comply with environmental regulations governing air emissions, wastewater discharge, solid waste disposal, and worker exposure to processing chemicals and dusts [61]. The European Union Industrial Emissions Directive (2010/75/EU) requires integrated permits covering all environmental media for facilities exceeding specified production capacities and mandates the application of Best Available Techniques to minimise ecological impacts [229].

Citrus biorefineries are typically not classified as intensive installations unless they are integrated with large-scale juice processing that exceeds the thresholds set by directives. However, individual member states may impose stricter requirements through national legislation [230]. Wastewater discharge from extraction processes requires treatment to meet quality standards for organic load, suspended solids, pH, and potentially specific pollutants, including residual solvents, before discharge to municipal sewers or receiving waters [10]. Air emissions from drying operations and solvent recovery systems may require control technologies, such as condensers, scrubbers, or thermal oxidisers, depending on emission rates and local air quality regulations [80].

Occupational safety regulations address worker exposure to processing chemicals, dust from dried citrus materials, and physical hazards, including high-temperature equipment, pressure vessels, and mechanical hazards [231]. The European Union REACH regulation requires registration of chemical substances manufactured or imported in quantities above specified thresholds, which may apply to highly purified biorefinery products marketed as distinct chemical substances rather than natural extracts [232]. Worker exposure limits for organic solvents, dust, and specific chemicals guide ventilation system design and personal protective equipment requirements, with compliance verification through workplace monitoring and health surveillance programmes [233].

### 5.3. Comprehensive Sustainability Assessment Frameworks

The development of standardised life-cycle assessment methodologies tailored explicitly for multi-product citrus biorefinery systems is a critical research priority. Current LCA studies exhibit substantial methodological heterogeneity regarding system boundaries, functional unit selection, allocation procedures, and impact assessment methods, precluding meaningful inter-study comparisons. Establishing consensus protocols through international working groups, potentially under ISO Technical Committee 207 or the European Platform on LCA, would enhance the comparability and credibility of environmental performance claims.

Integrating social LCA with environmental LCA and techno-economic analysis would enable a comprehensive sustainability evaluation that encompasses labour conditions, community impacts, health and safety considerations, and stakeholder acceptance [234]. Multi-criteria decision analysis frameworks that incorporate environmental, economic, social, and technical performance indicators can support a holistic comparison of alternative biorefinery configurations and inform evidence-based policy development.

Water–energy–food nexus considerations constitute an emerging research domain relevant to the sustainability assessment of citrus biorefineries. Lemon cultivation, processing, and biorefinery operations interact with water resources through irrigation, cleaning, extraction, and wastewater generation [235]. Energy is consumed throughout value chains, from agricultural inputs to industrial processing, while food security implications arise from land use, agricultural intensification, and the valorisation of processing residues. Integrated nexus assessments employing systems thinking approaches and computational modelling can identify trade-offs, synergies, and optimisation opportunities across interconnected resource systems [236].

### 5.4. Market Development and Value Chain Coordination

Consumer acceptance research on lemon biorefinery products remains underexplored, despite their critical importance to commercial success. Whilst technical feasibility and regulatory compliance are necessary prerequisites, market adoption ultimately depends on consumer perceptions, willingness to pay price premiums for sustainable products, and sensory acceptability [237]. Systematic consumer studies employing conjoint analysis, discrete choice experiments, and sensory evaluation panels can elucidate preference structures, identify optimal product positioning strategies, and quantify price-performance trade-offs for lemon-derived ingredients in food, cosmetic, and pharmaceutical applications [238].

Brand development and marketing communication strategies that specifically emphasise circular economic credentials, environmental benefits, and local sourcing represent underutilised value-creation opportunities. LCA results demonstrate that greenhouse gas emission reductions, water conservation, and waste diversion can be translated into consumer-facing ecolabels and sustainability certifications that differentiate lemon biorefinery products in crowded marketplaces. However, effective communication requires careful attention to avoid accusations of greenwashing, ensure claims are substantiated by credible third-party verification, and present information in accessible formats that resonate with target consumer segments.

Supply chain coordination mechanisms that can align incentives across diverse stakeholders—such as citrus growers, juice processors, biorefinery operators, ingredient purchasers, and end-product manufacturers—require a systematic investigation. Contract farming arrangements, cooperative ownership structures, and profit-sharing agreements can facilitate investment in biorefinery infrastructure whilst ensuring stable feedstock supplies and equitable value distribution. Applying supply chain management theories, including transaction cost economics, principal-agent frameworks, and relational contracting approaches, to the value chains of lemon biorefineries would elucidate governance structures conducive to long-term stability and mutual benefit.

### 5.5. Policy and Regulatory Research Needs

Regulatory science research addressing safety assessment protocols for novel lemon-derived ingredients represents a critical knowledge gap. Nanocellulose products, modified pectins, and concentrated bioactive extracts may require nanospecific toxicological evaluations, allergenicity assessments, and exposure modelling to meet regulatory requirements for food, pharmaceutical, and cosmetic applications. Proactive engagement with regulatory agencies—such as the FDA, EFSA, and competent national authorities—during product development stages can help identify data requirements, appropriate testing methodologies, and expedited approval pathways, thereby reducing time-to-market and regulatory compliance costs [226].

Policy effectiveness evaluations examining the impacts of circular-economy legislation, bioeconomy strategies, and waste-reduction targets on the development of citrus biorefineries would inform evidence-based policy refinement. Comparative analyses across jurisdictions with varying regulatory frameworks—such as the European Union’s Circular Economy Action Plan, California’s organic waste regulations, and China’s Circular Economy Promotion Law—can identify best practices, unintended consequences, and transferable policy instruments [239]. Ex-post assessments of implemented policies using econometric methods, case study analyses, and stakeholder consultations provide empirical foundations for adaptive policy management [240].

### 5.6. Concluding Remarks on Research Priorities

The research agenda delineated herein encompasses technological innovation, process optimisation, product development, sustainability assessment, market creation, and policy evolution. Priority should be given to research domains that offer substantial leverage for commercial implementation, including energy-efficient extraction and drying technologies, enzymatic bioconversion of flavonoid glycosides, reduction in nanocellulose production costs, integrated techno-economic and life-cycle assessments, and studies on consumer acceptance. Collaborative research partnerships spanning academia, industry, and government agencies are essential to mobilise the resources, expertise, and infrastructure needed to address the multifaceted challenges inherent in developing lemon biorefineries.

The successful realisation of commercial-scale lemon biorefineries that implement circular-economy principles will require sustained research investment, demonstration projects to validate integrated processing concepts, policy frameworks that incentivise sustainable practices, and market development initiatives to establish demand for lemon-derived bioproducts. The research priorities identified herein provide a strategic roadmap for advancing this transformative valorisation paradigm from technical possibility to industrial reality, thereby contributing meaningfully to global sustainability objectives whilst generating economic opportunities in lemon-producing regions worldwide.

### 5.7. Artificial Intelligence and Machine Learning in Lemon Biorefinery Optimisation

The integration of artificial intelligence (AI) and machine learning (ML) methodologies into biorefinery design and operation represents an emerging frontier with substantial potential to enhance process efficiency, economic performance, and sustainability outcomes [241]. Recent advances in computational approaches have demonstrated their applicability across multiple domains of agri-food waste biorefinery development, from predictive process modelling to supply chain optimisation and real-time process control [185,242].

In the domain of predictive modelling for extraction yields, supervised learning algorithms—including Artificial Neural Networks (ANN), random forest, and gradient boosting models (e.g., XGBoost)—have demonstrated superior predictive accuracy compared with conventional response surface methodology for optimising process parameters such as temperature, solvent ratio, extraction time, and power intensity in essential oil, pectin, and polyphenol recovery [243,244]. These data-driven models can capture non-linear interactions amongst process variables that polynomial regression models may inadequately represent, thereby enabling more precise optimisation of multi-response extraction systems. Furthermore, transfer learning approaches enable the adaptation of models trained on one citrus species to related feedstocks, reducing the experimental burden associated with process development for novel substrates [244].

Supply chain and feedstock management represent a second critical application domain. ML-based forecasting models can predict seasonal biomass availability, compositional variability, and quality parameters using agronomic, meteorological, and historical processing data, thereby enabling proactive inventory management and process parameter adjustments [242]. Optimisation algorithms for logistics network design—incorporating spatial distribution of feedstock sources, transportation costs, and processing capacity constraints—can identify cost-optimal collection routes and preprocessing facility locations. These tools are particularly valuable for cooperative biorefinery models that aggregate feedstock from multiple distributed suppliers.

Process monitoring and real-time control applications leverage soft-sensor technologies and digital-twin approaches to enable continuous operation of biorefineries. In anaerobic digestion systems, ML-based soft sensors can predict biogas yield, volatile fatty acid accumulation, and process stability in real time from readily measurable parameters (pH, temperature, electrical conductivity), thereby enabling early detection of D-limonene-mediated inhibition and proactive intervention [245,246]. Digital twin models that integrate process simulation with real-time sensor data offer the prospect of autonomous process optimisation, dynamically adjusting extraction parameters in response to changes in feedstock composition detected via near-infrared or Raman spectroscopy [185].

Finally, multi-objective optimisation using genetic algorithms and reinforcement learning can simultaneously balance economic returns, environmental impacts, and product portfolio selection across the entire biorefinery value chain. These approaches enable decision-makers to explore Pareto-optimal configurations that maximise cumulative revenue whilst minimising greenhouse gas emissions, water consumption, and energy demand—trade-offs that are difficult to resolve through conventional single-objective optimisation.

The convergence of AI/ML with *Industry 4.0* digital infrastructure, including Internet of Things (IoT) sensor networks, cloud computing, and edge analytics, positions the next generation of lemon biorefineries for substantially enhanced operational intelligence and adaptive process control. Readers are referred to recent comprehensive reviews on AI applications in agri-food waste biorefineries for detailed methodological guidance [185,242].

## 6. Conclusions

Building upon the extraction technologies and green processing methods established in Part I [6], this review has examined the implementation dimensions critical to translating laboratory advances into commercially viable lemon biorefinery operations. Integrated biorefinery design for valorising lemon-processing waste through cascade extraction represents a technically viable and environmentally beneficial pathway for converting disposal liabilities into diversified value streams.

Key findings show that cascade configurations that sequentially extract essential oils, polyphenols, pectin, and cellulosic materials consistently outperform single-product approaches. Cumulative revenues potentially exceed USD 400 per tonne of residue, whilst life-cycle assessments confirm greenhouse gas emission reductions of 60–85% and fossil resource depletion reductions of 70–95% relative to conventional disposal. These environmental benefits are maximised when facilities utilise renewable electricity or biogas cogeneration from anaerobic digestion of final residues. It is important to note, however, that the level of evidence underpinning these findings varies substantially across product streams. Essential oil extraction and conventional pectin recovery are commercially mature at TRL 8–9, with established industrial deployment across multiple facilities worldwide. Green extraction technologies, anaerobic digestion of de-oiled residues, and two-product integrated systems have been demonstrated at pilot scale (TRL 5–7), though widespread commercial adoption remains limited. By contrast, multi-product cascade configurations recovering five or more product streams, nanocrystalline cellulose production from citrus waste, and the cumulative revenue and IRR projections reported in Section 4 are derived primarily from techno-economic modelling and process simulation (TRL 3–5) and should be interpreted as economically promising estimates pending commercial-scale validation.

Economic viability remains scale-dependent. Small-scale processors handling fewer than 5000 tonnes annually face viability constraints, warranting cooperative models with centralised biorefinery facilities. Conversely, large integrated operations exceeding 100,000 tonnes annually achieve economies of scale supporting multi-product extraction with Internal Rates of Return exceeding 18% and payback periods under five years. Technology readiness varies substantially: essential oil extraction and pectin recovery have reached commercial maturity, whereas nanocrystalline cellulose production and platform chemical synthesis remain at pilot scale, necessitating phased implementation strategies.

Regulatory frameworks present distinct challenges across jurisdictions. In the European Union, novel lemon-derived products—particularly nanocrystalline cellulose and highly purified polyphenolic extracts—require Novel Food authorisation under Regulation (EU) 2015/2283, with average approval timelines of 2.6 years. In the United States, the GRAS framework offers faster market access but places greater responsibility on manufacturers to determine safety. Traditional products such as cold-pressed essential oils, citrus pectin at historical concentrations, and conventional citrus extracts generally qualify for exemptions under both regulatory systems, providing established revenue streams whilst novel products navigate approval processes.

The findings presented herein are broadly applicable across major lemon-producing regions—including the Mediterranean basin, Latin America, and South Asia—though optimal configurations will require contextual adaptation to local feedstock characteristics, energy costs, labour markets, and regulatory environments. Regional variations in peel composition (pectin content 20–30% DW, essential oil yield 0.8–2.55%) necessitate flexible processing designs that can accommodate seasonal and varietal fluctuations.

Ultimately, transforming lemon processing waste from an environmental liability into a valuable resource depends on sustained collaboration among researchers, processors, policymakers, and financial institutions. Whilst technical feasibility and economic potential have been demonstrated, translating this potential into widespread commercial reality represents the essential next phase in advancing circular bioeconomy transitions within the global citrus industry. Priority actions include demonstration projects to validate integrated processing at scale, harmonised regulatory guidance for biorefinery products, and market development initiatives to establish robust demand for lemon-derived bioproducts.

## Figures and Tables

**Table 1:** Optimal Operational Parameters for Anaerobic Digestion of Lemon Processing Residues.

Parameter	Mesophilic Single Stage	Thermophilic Single Stage	Stage System	Ref.
Operating Temperature (°C)	37–42	51–55	55 (Stage 1) 37 (Stage 2)	[116,120]
Optimal OLR ^1^ (g VS·L^−1^·day^−1^)	1.00	0.600	1.20	[117,120]
Maximum Essential Oil Load (mg·L^−1^·day^−1^)	47.60	35.20	88.10	[120]
Methane Yield (L CH_4_·(g VS)^−1^)	0.46	0.12	0.52	[116,120]
Methane Content (% vol.)	55–62	48–55	58–65	[118]
Hydraulic Retention Time (days)	30–40	25–35	35 (total)	[116,123]
Process Stability	Good	Moderate	Excellent	[116]
Inhibition Threshold (g VS·L^−1^·day^−1^)	1.98	1.50	2.50	[120]

^1^ OLR: Organic Loading Rate; VS: Volatile Solids.

**Table 2:** Life Cycle Assessment (LCA) Impact Categories for Lemon Biorefinery Scenarios.

Impact Category	Baseline Scenario (Conventional)	Biorefinery with Biogas Sale	Biorefinery with Biogas Recirculation	Ref.
Global Warming Potential (kg CO_2_ eq per tonne)	450	280	95	[129]
Cumulative Energy Demand (MJ per tonne)	3800	2400	1550	[129]
Acidification Potential (kg SO_2_ eq per tonne)	2.8	2.1	1.2	[47]
Eutrophication Potential (kg PO_4_ eq per tonne)	0.65	0.42	0.28	[45]
Photochemical Oxidation (kg C_2_H_4_ eq per tonne)	0.15	0.11	0.07	[68]
Terrestrial Ecotoxicity (kg 1,4-dichlorobenzene (1,4-DB) eq per tonne)	18.5	12.3	8.4	[45]

**Table 3:** Comparative Techno-Economic Parameters for Lemon Biorefinery Configurations.

Parameter	Small Scale (25,000 Tonnes per Year)	Medium Scale (100,000 Tonnes per Year)	Large Scale (400,000 Tonnes per Year)	Ref.
Capital Investment (Million USD) ^1^	3.7	9.6	17.8	[11]
Ethanol Production Cost (USD per litre) ^1^	3.80	1.34	0.68	[11]
Internal Rate of Return (%)	12–15	16–19	20–24	[131]
Transportation Cost Sensitivity (USD per litre of ethanol) ^2^	High (+0.51)	Moderate (+0.25)	Low (+0.11)	[11]
Minimum Pectin Price for Viability (USD per kilogram)	12	8	6	[2]

^1^ inflation-adjusted to 2025; ^2^ at a rate of USD 10 per tonne transported.

**Table 4:** Significant Scalability Challenges and Potential Solutions in Lemon Biorefinery Implementation.

Challenge Category	Specific Issues	Potential Solutions	Ref.
Process Scale-Up	Energy-intensive extraction and separation processes; solvent recovery demands up to 73% of biomass energy content	Implementation of heat integration strategies; adoption of low-energy separation technologies; utilisation of residual biomass for process heat generation	[135,145]
Feedstock Management	Seasonal availability of lemon processing residues; rapid quality deterioration due to microbial activity; inconsistent chemical composition	Establishment of efficient cold-chain logistics; implementation of short-term preservation methods (cooling, grinding, drying, pH adjustment); development of flexible processing schedules; feedstock standardisation protocols	[62,143]
Capital Investment	High initial capital expenditure (CAPEX) on specialised equipment; biorefinery equipment costs represent 57% of total capital requirements	Modular biorefinery designs enabling phased implementation; co-location with existing citrus processing facilities to share infrastructure; public–private partnerships and green financing mechanisms	[2,134]
Technology Maturity	Variable technological readiness levels across valorisation pathways; nanocellulose and bioactive extraction at pilot scale	Prioritised commercialisation of mature technologies (essential oils, pectin); sustained R&D investment in emerging pathways; establishment of demonstration facilities	[143,147]
Market Establishment	Absence of established demand channels for novel bioproducts; limited market awareness of lemon-derived ingredients	Development of strategic partnerships with end-user industries; marketing campaigns emphasising sustainability and performance benefits; participation in industry trade shows and technical forums	[135]
Process Integration	Complexity of coordinating sequential extraction steps; optimisation of multi-product biorefineries	Implementation of cascade biorefinery configurations; process simulation and optimisation using advanced software tools; adoption of Industry 4.0 technologies for real-time monitoring	[2,45]

**Table 5:** Regulatory Requirements for Lemon Biorefinery Products in Major Markets.

Product Category	United States	European Union	Key Specifications	Typical Approval Timeline
Essential Oils/ D-limonene	GRAS status under 21 CFR Part 582; mandatory notification under revised GRAS rule (2025)	Approved flavouring substance under Regulation EC No. 1334/2008; compliance with purity specifications	Minimum 95% D-limonene purity for food-grade; heavy metal limits (Pb < 2 ppm); peroxide value specifications	6–12 months (notification pathway)
Pectin (HM and LM)	Food additive status: compliance with Food Chemicals Codex specifications	Codex Alimentarius INS 440; compliance with EU purity criteria (Directive 2008/84/EC)	Galacturonic acid content ≥ 65%; degree of esterification specifications; microbiological limits	3–6 months (established ingredient)
Bioactive Compounds (Flavonoids)	Novel food ingredient requiring GRAS notification with comprehensive safety data	Novel food authorisation through EFSA; extensive toxicological evaluation required	Identity and purity specifications; absence of genotoxicity; acceptable daily intake determination	18–36 months (EFSA evaluation)
Nanocellulose (CNC/CNF)	Nanomaterial-specific risk assessment required; potential food contact substance notification	Compliance with Recommendation 2011/696/EU on nanomaterial definition; nano-specific safety assessment	Particle size characterisation; surface chemistry analysis; migration testing for food contact applications	24–48 months (comprehensive evaluation)
Citric Acid	GRAS status for food-grade applications; USP specifications for pharmaceutical grade	Approved food additive E330; compliance with EU specifications for food-grade material	Assay ≥ 99.5% (anhydrous); clarity of solution; heavy metal limits	3–6 months (established ingredient)

**Table 6:** Techno-Economic Indicators for Integrated Lemon Biorefinery Systems.

Performance Indicator	Value Range	Optimal Conditions	Critical Success Factors	Ref.
Minimum Processing Scale for Economic Viability	360–500 tonnes of dry peel per year	≥500 tonnes per year for positive NPV	Secured long-term feedstock supply contracts; established markets for multiple co-products	[134,135]
Capital Expenditure (CAPEX)	USD 15–35 million for a 500 tonne per year facility	Optimised through colocation with existing citrus processing	Integration with existing infrastructure; modular design implementation; equipment standardisation	[2]
Operating Expenditure (OPEX)	USD 250–450 per tonne of dry peel processed	Process integration to minimise energy costs; efficient solvent recovery	Energy efficiency improvements; automation of labour-intensive steps; economies of scale in chemical procurement	[147]
Revenue Potential	USD 400–650 per tonne of dry lemon peel	Diversified product portfolio including essential oils, pectin, bioactive compounds, and nanocellulose	Achieving premium product quality, securing high-value market segments, and sustainability certification	[164]
Energy Efficiency	25–45% of the biomass energy content is consumed in processing	<30% through heat integration and residual biomass combustion	Implementation of energy recovery systems; optimised separation technologies; combined heat and power generation	[145]
Greenhouse Gas Emissions	0.9–1.5 kg CO_2_-eq per kg of products	<1.0 kg CO_2_-eq through optimised process design	Renewable energy utilisation; efficient solvent recovery; avoided emissions from waste disposal	[68,145]
Water Consumption	8–15 litres per kg of dry peel	<10 litres through water recycling	Implementation of closed-loop water systems; membrane-based separation technologies	[45,57]
Payback Period	5–9 years	<7 years with favourable financing	Rapid market penetration; achievement of design capacity; stable feedstock and product prices	[165]
Internal Rate of Return (IRR)	8–18%	>15% for an attractive investment	Diversified revenue streams; operational excellence; sustainability premium pricing	[165]

**Table 7:** Market Value and Growth Projections for Lemon Biorefinery Products (2024–2035).

Product	Global Market Value 2024 (Million USD)	Projected Market Value 2030–2035 (Million USD)	CAGR (%)	Primary Applications	Key Market Drivers	Ref.
D-limonene	351	469–559 (2032–2035)	5.1–6.0	Food flavouring (52%), cosmetics and personal care (25%), industrial cleaning (15%)	Consumer preference for natural ingredients, regulatory phase-out of synthetic solvents, and the expanding aromatherapy market	[155]
Lemon Essential Oil	450	600 (2029)	5.0	Aromatherapy and wellness products, the fragrance industry, and pharmaceutical applications	Growing health and wellness sector; scientific validation of therapeutic properties; premium positioning in the natural products market	[164]
Citrus Pectin	1300	2100–2300 (2033–2035)	5.5–7.0	Food and beverages (62%), pharmaceuticals (18%), personal care (8%)	Increasing demand for clean-label food additives, expansion of reduced-sugar food products, and pharmaceutical applications in drug delivery	[168,169]
Nano- cellulose (CNC/CNF)	490–580	2260–3160 (2033–2034)	18.5–20.1	Packaging (31%), composites (24%), biomedical applications (18%), electronics (12%)	Sustainability mandates driving bio-based material adoption; superior mechanical properties; emerging applications in advanced materials	[162]
Bioactive Compounds (Flavonoids)	180–250 (estimated)	400–550 (2033)	8.5–10.0	Dietary supplements, functional foods, and pharmaceutical formulations	Increasing consumer awareness of health benefits, scientific evidence supporting therapeutic efficacy, and the growing nutraceutical sector	[170,171]
Citric Acid	3500 (total market; lemon-derived represents <5%)	4200–4500 (2030)	4.0–5.0	Food and beverages (60%), pharmaceuticals (18%), industrial applications (15%)	Growing food processing industry, pharmaceutical excipient demand, and industrial chelating agent applications	[166,167]

## Data Availability

The original contributions presented in this study are included in the article. Further inquiries can be directed to the corresponding authors.

## Abbreviations

The following abbreviations are used in this manuscript:

AD	Anaerobic Digestion
AI	Artificial Intelligence
ANN	Artificial Neural Networks
AP	Acidification Potential
B2B	Business-to-Business
B2C	Business-to-Consumer
CAGR	Compound Annual Growth Rate
CAPEX	Capital Expenditure
CED	Cumulative Energy Demand
CHP	Combined Heat and Power
CNC	Cellulose Nanocrystals
CNF	Cellulose Nanofibrils
DES	Deep Eutectic Solvents
DW	Dry Weight
EAE	Enzyme-Assisted Extraction
EFSA	European Food Safety Authority
EO	Essential Oil
EP	Eutrophication Potential
FDA	Food and Drug Administration (United States)
GRAS	Generally Recognized as Safe
GWP	Global Warming Potential
HM	High Methoxyl
IGP	Indicazione Geografica Protetta (Protected Geographical Indication)
INS	International Numbering System
IoT	Internet of Things
IRR	Internal Rate of Return
ISO	International Organization for Standardization
LCA	Life Cycle Assessment
LM	Low Methoxyl
MAE	Microwave-Assisted Extraction
MCC	Microcrystalline Cellulose
ML	Machine Learning
NCC	Nanocrystalline Cellulose
NFC	Nanofibrillated Cellulose
NPV	Net Present Value
OLR	Organic Loading Rate
OPEX	Operating Expenditure
PBP	Payback Period
PDO	Protected Designation of Origin
PEF	Pulsed Electric Field
PLA	Polylactic Acid
RSM	Response Surface Methodology
SC-CO_2_	Supercritical Carbon Dioxide
SCP	Single-Cell Protein
SFE	Supercritical Fluid Extraction
SME	Small and Medium Enterprise
SSF	Solid-State Fermentation
TEA	Techno-Economic Analysis
TRL	Technology Readiness Level
UAE	Ultrasound-Assisted Extraction
UAEE	Ultrasound-Assisted Enzymatic Extraction
UASB	Upflow Anaerobic Sludge Blanket
USP	United States Pharmacopoeia
VS	Volatile Solids
WTP	Willingness to Pay
